# Plant Cytosolic Ascorbate Peroxidase with Dual Catalytic Activity Modulates Abiotic Stress Tolerances

**DOI:** 10.1016/j.isci.2019.05.014

**Published:** 2019-05-15

**Authors:** Dan-Chu Chin, Rajendran Senthil Kumar, Ching-Shu Suen, Chia-Yu Chien, Ming-Jing Hwang, Chun-Hua Hsu, Xu Xuhan, Zhong Xiong Lai, Kai-Wun Yeh

**Affiliations:** 1 Institute of Plant Biology, National Taiwan University, Taipei 106, Taiwan; 2 Institute of Biomedical Sciences, Academia Sinica, Taipei 115, Taiwan; 3 Department of Agricultural Chemistry, National Taiwan University, Taipei 106, Taiwan; 4 Institute of Horticultural Biotechnology, Fujian Agriculture and Forestry University, Fuzhou, China

**Keywords:** Plant Biochemistry, Plant Biology, Plant Evolution

## Abstract

Ascorbic acid-glutathione (AsA-GSH) cycle represents important antioxidant defense system in planta. Here we utilized *Oncidium* cytosolic ascorbate peroxidase (*OgCytAPX*) as a model to demonstrate that *CytAPX* of several plants possess dual catalytic activity of both AsA and GSH, compared with the monocatalytic activity of *Arabidopsis* APX (*AtCytAPX*). Structural modeling and site-directed mutagenesis identified that three amino acid residues, Pro^63^, Asp^75^, and Tyr^97^, are required for oxidization of GSH in dual substrate catalytic type. Enzyme kinetic study suggested that AsA and GSH active sites are distinctly located in cytosolic APX structure. Isothermal titration calorimetric and UV-visible analysis confirmed that cytosolic APX is a heme-containing protein, which catalyzes glutathione in addition to ascorbate. Biochemical and physiological evidences of transgenic *Arabidopsis* overexpressing *OgCytAPX1* exhibits efficient reactive oxygen species-scavenging activity, salt and heat tolerances, and early flowering, compared with *Arabidopsis* overexpressing *AtCytAPX*. Thus results on dual activity *CytAPX* impose significant advantage on evolutionary adaptive mechanism in planta.

## Highlights

• Cytosolic ascorbate peroxidase in several plants possess two substrate oxidation
• Amino acid residues Pro63, Asp75, and Tyr97 were identified as GSH oxidation
• OgcytAPX1-catalyzing GSH is an independent biochemical step in AsA-GSH cycle
• *CytAPX*s with two substrate-binding activities enhance stress tolerance and flowering

## Graphical Abstract

**Figure:**
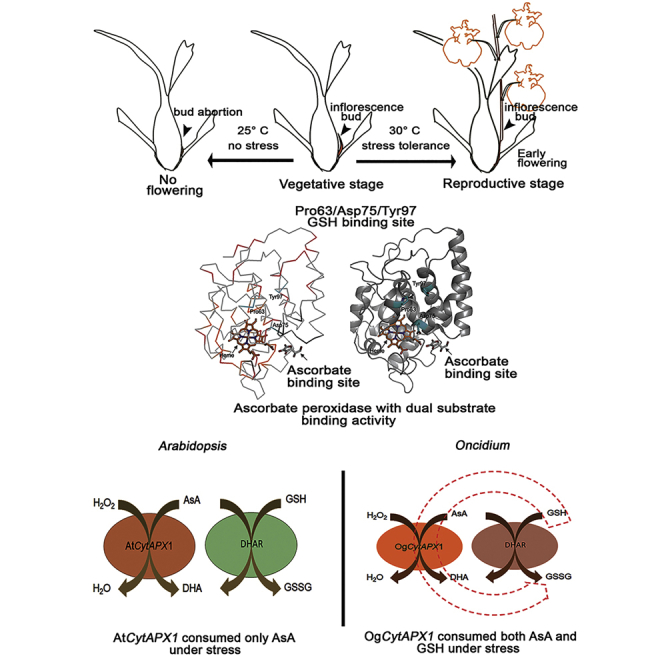

## Introduction

Plants generate reactive oxygen species (ROS) continuously as by-products of various metabolic pathways and stresses in different cell compartments. Several antioxidants are usually employed by plants to eliminate the oxidative damage from ROS under various growth and stress conditions (Suzuki et al., 2012). The ascorbate (AsA)-glutathione (GSH) cycle is an essential metabolic pathway for the detoxification of ROS and regulation of the cellular level of H_2_O_2_ (Foyer and Shigeoka, 2011). The pathway contains ascorbate peroxidase (APX) together with dehydroascorbate reductase (DHAR) and glutathione reductase (GR), in addition to antioxidant metabolites AsA, GSH, and NADPH. AsA, GSH, and NADPH form redox couples with different redox potential and concentration and play the important role of maintaining redox homeostasis in plants to protect them from oxidation damage (Foyer and Noctor, 2016). APX enzymes (EC1.11.1.11) are class I heme peroxidases and catalyze the electron transfer from AsA to scavenge H_2_O_2_. In plant cells, AsA is the most important reducing substrate for H_2_O_2_ detoxification, with the oxidized product being dehydroascorbate (DHA). DHA is reduced to AsA by the action of DHAR, which uses GSH as the reducing substrate, and subsequently generates glutathione disulfide (GSSG). GSSG is in turn re-reduced to GSH by the catalysis of GR using NADPH as electron donor (Sharma et al., 2012). GSH is a non-protein thiol metabolite with a tripeptide (γ-glu-cys-gly) structure. The fundamental function of GSH is in thiol-disulfide interactions, in which reduced GSH is interchangeable with the oxidized form, GSSG (Frendo et al., 2013). Both AsA and GSH are ubiquitous in eukaryotic organisms, but only AsA is specific and highly abundant in plants, where it is essential for growth and development (Foyer and Noctor, 2016).

The AsA level and redox state have been reported to play a role in cell proliferation and elongation (Gest et al., 2013, Pignocchi and Foyer, 2003) and flowering (Chin et al., 2014, Kotchoni et al., 2009). The role of APX isoforms in overcoming various environmental stresses has been reviewed recently (Pandey et al., 2017). Knockout APX-1 mutants in *Arabidopsis* are sensitive to both drought and heat stress, resulting in increased Calvin cycle enzymes without changing the amount of glycerate-3-phosphate and ribulose-5-phosphate (Koussevitzky et al., 2008). Likewise, APX is more sensitive to heavy metals in double-silenced APX1 and APX2 transgenic rice plants, which displayed normal growth and enhanced tolerance (Rosa et al., 2010). In comparison, the GSH redox system has been implicated in the regulation of cell death (De Pinto et al., 2012), root development, and meristem differentiation (Bashandy et al., 2010, Yu et al., 2013). Reports have been published on the association between flowering and GSH levels or GSH biosynthetic rates in *Arabidopsis* and *Eustoma grandiflorum* (Hatano-Iwasaki and Ogawa, 2012, Ogawa, 2005, Yanagida et al., 2004). Both AsA and GSH are highly reduced under optimal conditions. However, the compounds shift toward a more oxidized state in response to increases in intracellular ROS, suggesting that the changes in AsA/GSH redox status under oxidative stress have the ability to trigger several processes in development and growth, including phase transition and flowering initiation.

Our recent work has indicated that *Oncidium* orchid grown under prolonged high ambient temperature exposure (at 30°C lasting for 14 days) is induced to early flowering (Chin et al., 2014). Investigation of the flowering mechanism revealed that *CytAPX1* gene expression and enzymatic activity are increased by prolonged high-temperature exposure, which leads to decreased AsA level or AsA redox ratio, as well as decrease of GSH level or GSH redox ratio (Chin et al., 2016). Moreover, transgenic *Arabidopsis* ectopically overexpressing *Oncidium CytAPX1* displayed an early-flowering phenotype, accompanied by low level of H_2_O_2_ and low AsA redox ratio under 30°C growth condition. This suggests that *CytAPX1*-mediated AsA and GSH redox homeostasis is a critical factor for the mechanism of flowering induction against prolonged high ambient temperature. This observation prompted us to characterize the physiological, biochemical, and molecular functions of *Oncidium CytAPX1*. In the current study, we found that *Oncidium* cytosolic APX1 (*OgCytAPX1*) and those in some plant species can catalyze not only AsA but also GSH as electron donors to scavenge H_2_O_2_. Enzyme kinetic analysis suggested that two distinct active sites are present for binding AsA and GSH, respectively, in *OgCytAPX1*. Data from structural modeling revealed that a possible GSH-binding site composed of Pro^63^, Asp^75^, and Tyr^97^, in addition to AsA-binding site, was identified in *OgCytAPX1*, whereas in *Arabidopsis* the corresponding site is composed of Asp^63^, His^75^, and His^97^ without GSH-binding activity. Ultraviolet-visible (UV-vis) analysis and isothermal titration calorimetry confirmed that *OgCytAPX* uses heme group to catalyze GSH. When *OgCytAPX1* and *AtCytAPX1* were overexpressed in *Arabidopsis* Col-0, only *OgCytAPX1*-OE plants showed a significant reduction in H_2_O_2_ level and GSH redox ratio, thus resulting in earlier flowering. Therefore our finding validates that the *CytAPX*s of several plants possessing APX/glutathione peroxidase (GPX) activities with two substrate recognition sites of oxidizing AsA and GSH in plants being functional to enhance stress tolerance and modulate flowering initiation and environmental adaption.

## Results

### In-Gel Assays Illustrate *OgCytAPX* Possesses Dual Substrate-Binding Activities for Ascorbate and Glutathione

**Figure 1:**
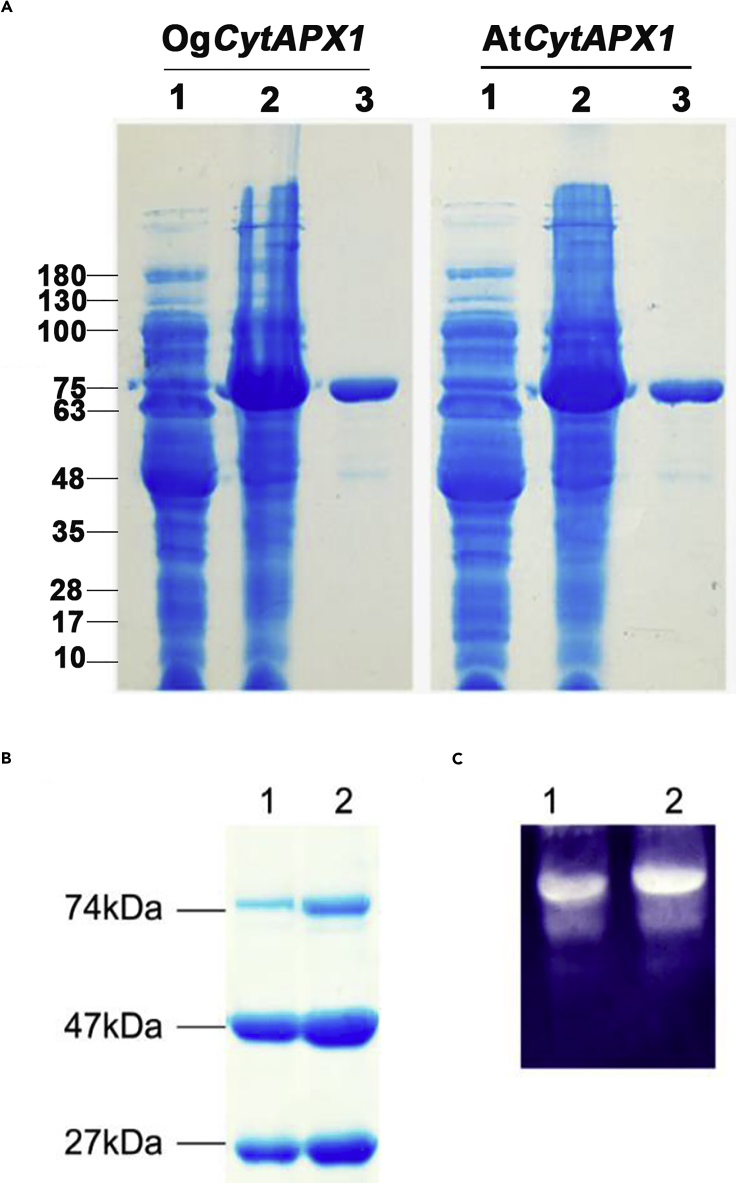
Polyacrylamide Gel Electrophoresis Analysis of Recombinant Cytosolic Ascorbate Peroxidase 1 (*CytAPX1*) Proteins of *Oncidium* and *Arabidopsis* (A) Total protein extracts of *Oncidium OgCytAPX1* (left panel) and *Arabidopsis AtCytAPX1* (right panel) expressed in *E. coli*; lane 1, non-isopropyl β-thiogalactopyranoside (IPTG) induction (40 μg); lane 2, 0.1 mM IPTG induction (40 μg); lane 3, purified fusion protein, MBP-*CytAPX1* (10 μg). (B) Purified recombinant MBP-*CytAPX* (30 μg) after factor Xa digestion; fusion protein (74 kDa), MBP tag (47 kDa), free *CytAPX1* (27 kDa); lane 1 for *Oncidium* and lane 2 for *Arabidopsis*. (C) *In-gel* staining for assaying recombinant APX activity. Digested recombinant protein (50 μg) was assayed on native polyacrylamide gel electrophoresis (10%); lane 1 for *Oncidium* and lane 2 for *Arabidopsis*.

*OgCytAPX1* was cloned from mRNA transcripts of pseudobulb tissues using RT-PCR. Sequence analysis predicted 250 amino acid residues with approximate molecular mass 27 kDa. Recombinant protein was produced in *E. coli* BL21 (Codon Plus) by cloning the *OgCytAPX1* gene in pMAL-c5x vector, as described in methods. *AtCytAPX1* from *Arabidopsis* was also produced for comparing experiment. Both fusion proteins linked with an MBP tag (maltose-binding protein) were expressed with approximate molecular weight 74 kDa (Figure 1A). After purification and digestion by protease factor Xa to remove the MBP tag, an approximate 27-kDa target protein was obtained (Figure 1B). Both *OgCytAPX1* and *AtCytAPX1* recombinant proteins showed functional APX activity by *in-gel* activity assays (Figure 1C).

**Figure 2:**
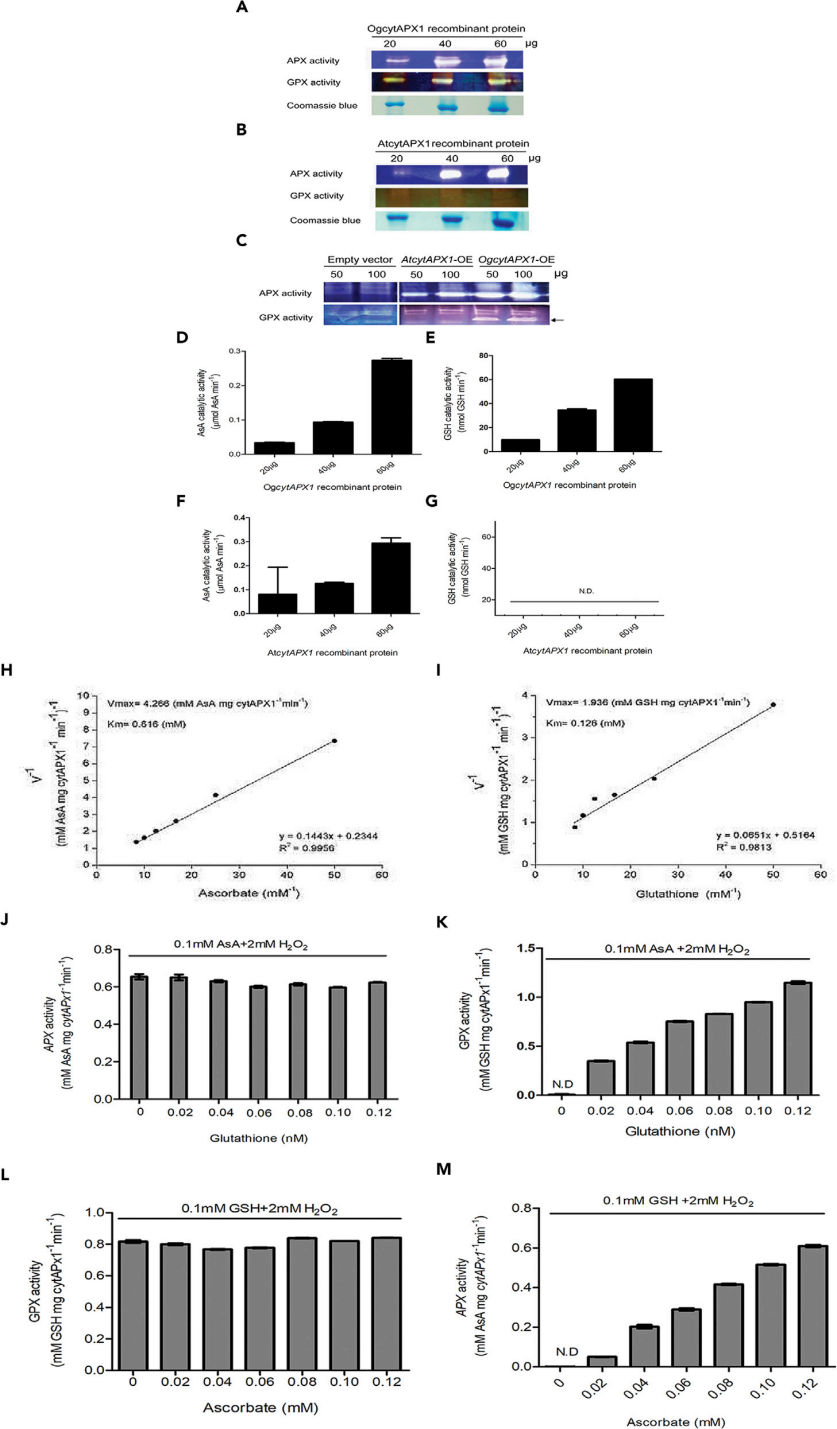
The Activity and Kinetic Assay of APX and GPX in *OgCytAPX1* and *AtCytAPX1* Proteins (A and B) Purified and free recombinant proteins (~27 kDa, expressed by *E. coli*) of 20, 40 and 60 μg were resolved on native-PAGE (10%), then assayed for APX (upper gel) and GPX activity (middle gel) respectively. Coomassie Blue staining used as internal control (bottom gel). (C) *OgCytAPX1* proteins overexpressed in *Arabidopsis* were extracted and assayed for APX (upper gel) and GPX (lower gel) activities. The arrow indicated that overexpressed *OgCytAPX1* showed strong GPX activity. Empty vector expression used as negative control. *OgCytAPX1*-OE and *AtCytAPX1*-OE indicate *Arabidopsis* plants overexpressing *Oncidium CytAPX1* or *Arabidopsis CytAPX1*. (D and E) Enzymatic activities of APX and GPX of the recombinant *OgCytAPX1* protein, respectively. (F and G) Enzymatic activities of APX and GPX activities of recombinant *AtCytAPX1* protein, respectively. (H and I) Kinetic assay of *OgCytAPX1* for APX and GPX activity, respectively. (J and K) Varied concentration of GSH in APX and GPX activities of *OgCytAPX1*, respectively. (L and M) The concentration-dependent AsA in GPX and APX activities of *OgCytAPX1*. 0.1mM AsA or GSH and 2 mM H_2_O_2_ were loaded for enzymatic reaction of APX and GPX. Each loading sample was 50 μg purified recombinant *OgCytAPX1* protein. Error bar indicates SEM (n = 5).

The *OgCytAPX1* and *AtCytAPX1* recombinant proteins were further assayed *in vitro* to determine substrate-oxidizing ability. *OgCytAPX1* displayed oxidizing activities for both substrates, indicating possession of both APX and GPX activities, whereas *AtCytAPX1* displayed only APX activity (Figures 2A, 2B, and 2D–2G). Further confirmation of substrate-oxidizing activity was obtained by overexpressing *OgCytAPX1* and *AtCytAPX1* in *Arabidopsis*. Crude proteins extracted from transgenic *Arabidopsis* lines were used for in-gel activity assay. As shown in Figure 2C, both *Arabidopsis* lines overexpressed *CytAPX1* as demonstrated by the high intensity of the band in the APX in-gel activity assay. Moreover, crude proteins extracted from *OgCytAPX1*-OE plants displayed a band of GPX activity in addition to APX activity. In contrast, protein samples extracted from *AtCytAPX1*-OE and empty vector plants showed no signals in GPX activity. Enzyme kinetic assay to determine the Lineweaver-Burk plot was performed (Figures 2H and 2I). The reaction rate of *OgCytAPX1* against AsA substrate was Km 0.616 (mM) and Vmax 4.266 (mM AsA mg CytAPX1^−1^ min^−1^), whereas against GSH was Km 0.126 (mM) and Vmax 1.936 (mM GSH mg CytAPX1^−1^ min^−1^). The result suggested that the binding affinity of *OgCytAPX1* to GSH is higher than to AsA. To clarify whether the active sites on *OgCytAPX1* for binding AsA and GSH are distinct, the substrate-binding competition assay, by adding AsA and GSH together to react with *OgCytAPX1*, was performed. As shown in Figure 2J, the varied GSH concentration from 0.02 to 0.12 mM did not interfere with the APX activity on AsA (with 0.1 mM constant concentration). However, at the same constant AsA concentration (0.1 mM), GPX activity of *OgCytAPX1* linearly increased with increasing GSH concentration from 0.02 to 0.12 mM (Figure 2K). Likewise, GPX activity of *OgCytAPX1* on GSH was not interfered by AsA (Figure 2L). Moreover, APX activity of *OgCytAPX1* was identical in Michaelis-Menten behavior at varied concentration of AsA, even at 0.1mM GSH (Figure 2M). The data ruled out the possibility of substrate-binding competition between AsA and GSH toward *OgCytAPX1*. It suggested that *OgCytAPX1* possessed two distinct active sites for AsA and GSH. Therefore it exerts APX and GPX activities independently.

### Identification of the GSH-Binding Site by Structural Modeling and Site-Directed Mutagenesis

**Figure 3:**
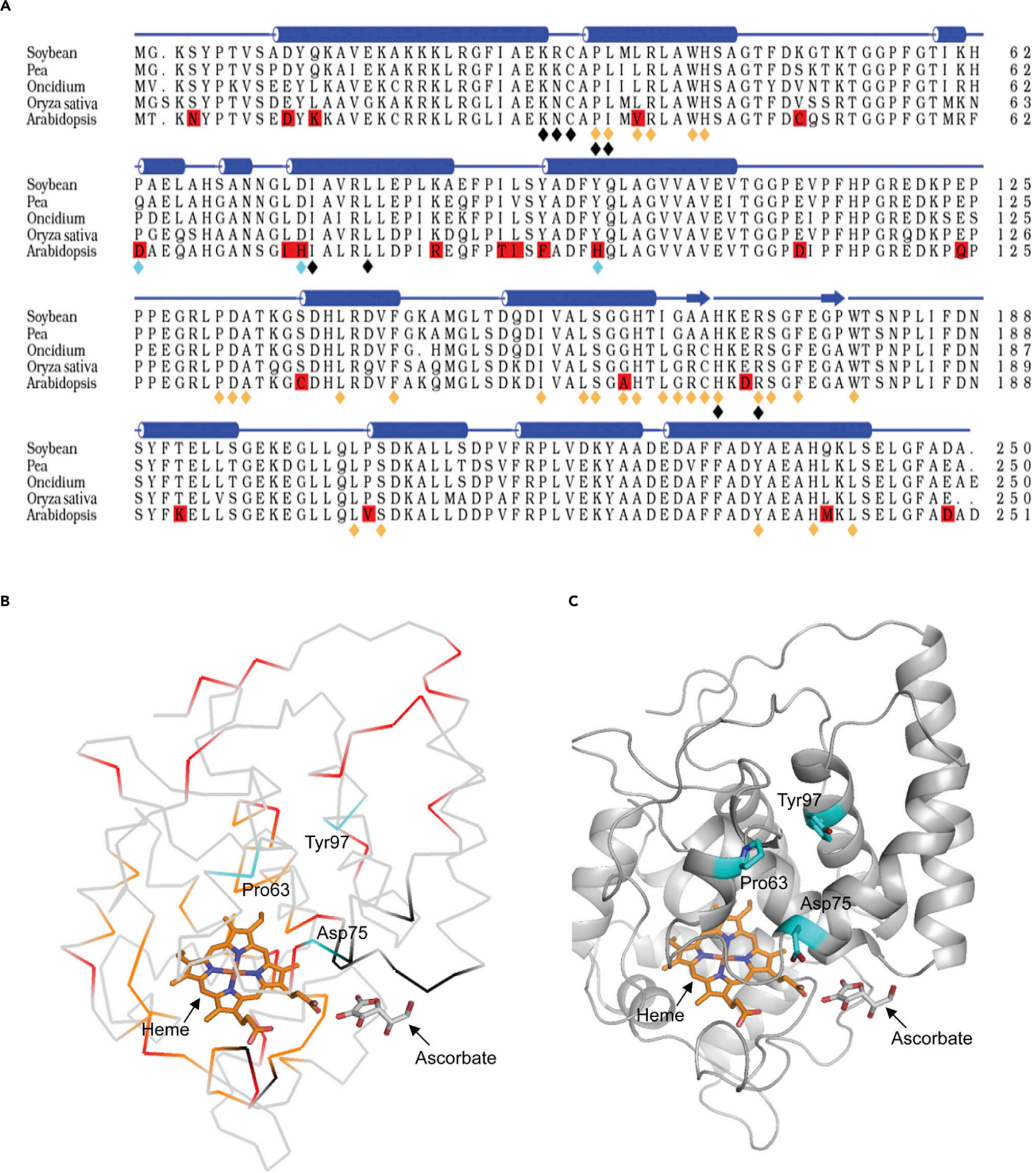
Sequence Alignment of *OgCytAPX1* with Some Related Plant APXs, and the Sequence Homology-Based Predicted Three-Dimensional Structures (A) Sequence alignment was performed with the program ClustalW (Larkin et al., 2007). The final figure was prepared using Alscript (Barton, 1993). The secondary structure elements on the top of the alignment are based on the crystal structure of soybean *CytAPX1* (PDB: 1OAF) (Sharp et al., 2003). The blue bars represent the secondary structure, cylinders are α-helices, bars with arrows are β-sheets, and lines are coil/loop; 22 amino acid residues marked by red background indicate the difference between *Oncidium* (or *Oryza sativa*) and *Arabidopsis*. The conserved residues corresponding to heme binding and AsA binding in the crystal structure of soybean *CytAPX1* are marked by orange and black diamonds, respectively. Cyan diamonds represent Proline (Pro) 63, Aspartate (Asp) 75, and Tyrosine (Tyr) 97, required for GSH oxidation activity in *Oncidium.* (B) C-alpha atom trace form or mode of *OgCytAPX1*. The homology modeling structure of *OgCytAPX1* using the 3D coordination of soybean *CytAPX1* (PDB: 1OAF) as the template. The modeling process was carried out using Modeler/Discovery Studio (Accelrys Inc., San Diego, CA, USA). Figures were generated by PyMol (http://pymol.sourceforge.net.). Ligand sites for heme and AsA were adopted from the template. Heme-binding residues were marked in orange, and AsA-binding residues were marked in black. Residues differing between *Oncidium* (or *Oryza sativa*) and *Arabidopsis* were in red. The three residues Pro63, Asp75, and Tyr 97 that were mutated in this study were marked by cyan carbon atoms in stick mode. (C) C-alpha atom ribbon form or mode of *OgCytAPX1*. The three residues Pro63, Asp75, and Tyr97 required for GSH oxidization are marked in cyan. The structure shows the location of GSH, AsA, and heme-binding sites.

**Figure 4:**
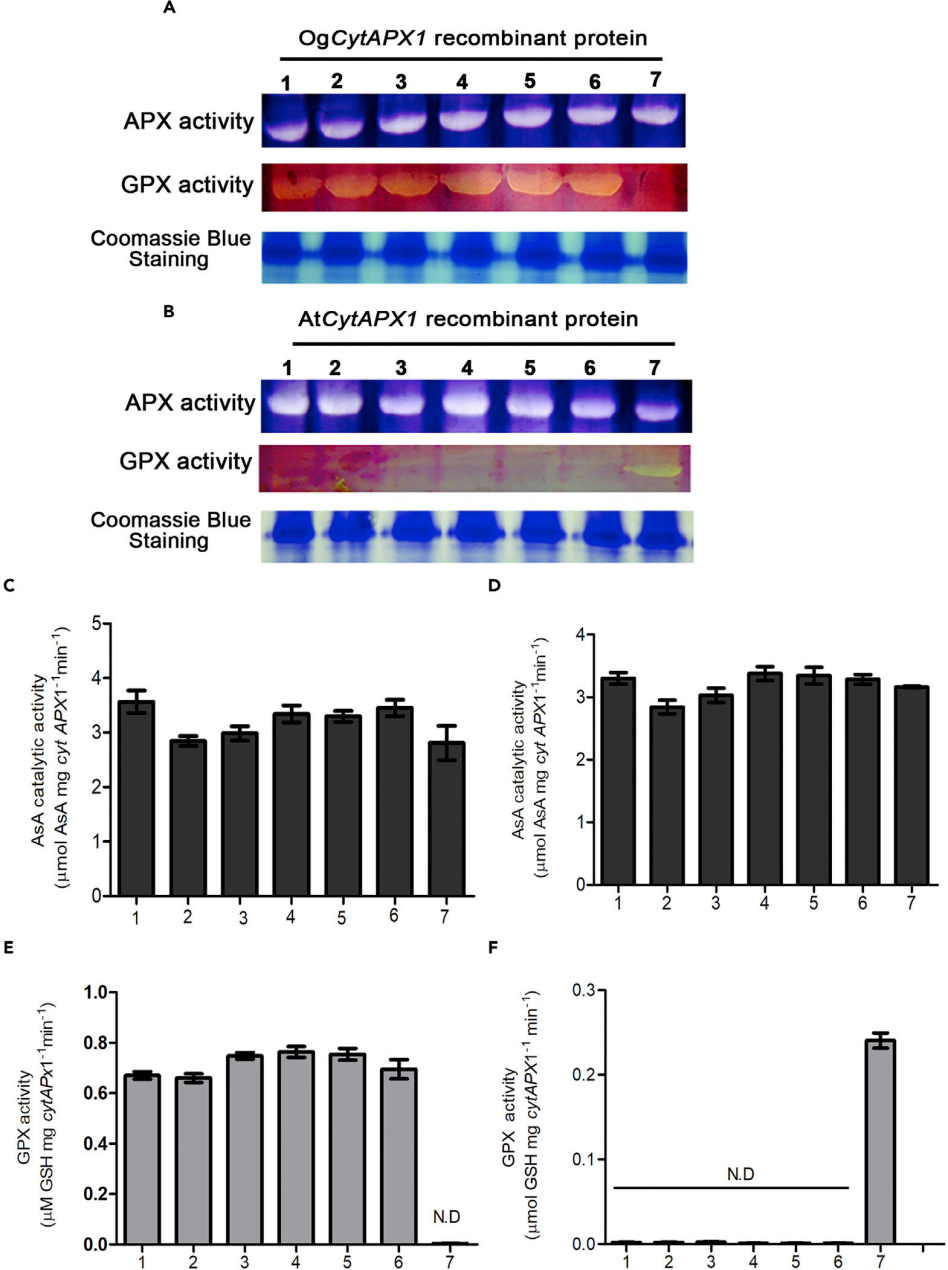
APX and GPX Activity Assay of Various Mutant Proteins of *OgCytAPX1* and *AtCytAPX1* (A) Purified free recombinant proteins of *OgCytAPX1* (∼27 kDa) with single-residue mutant: Pro63Asp (lane 1), Asp75His (lane 2), and Tyr97His (lane 3); double-residue mutants: Pro63Asp-Asp75His (lane 4), Pro63Asp-Tyr97His (lane 5), and Asp75His-Tyr97His (lane 6); and triple-residue mutant: Pro63Asp-Asp75His-Tyr97His (lane7) were resolved on 10% native gel and then assayed for APX activity (upper gel) and GPX activity (middle gel). (B) Purified free recombinant proteins *AtCytAPX1* (∼27 kDa) and its derived mutants, including with single-residue mutation: Asp63Pro (lane 1), His75Asp (lane 2), and His97Tyr (lane 3); double-residue mutation: Asp63Pro-His75Asp (lane 4), Asp63Pro-His97Tyr (lane 5), and His75Asp-His97Tyr (lane 6); and triple-residue mutation: Asp63Pro-His75Asp-His97Tyr (lane 7), were resolved on 10% native gel and then assayed for APX activity (upper gel) and GPX activity (middle gel). Coomassie blue staining was used as internal control (bottom gel). (C and D) APX activities of the purified OgCytAPX1 and AtCytAPX1 recombinant proteins were measured by the associated histogram analysis, respectively. (E and F) GPX activities of the purified OgCytAPX1 and AtCytAPX1 recombinant proteins were measured by the associated histogram analysis, respectively. Each loading sample was 50 μg purified free recombinant protein.

To dissect the structural site for GSH binding, alignment of amino acid sequence among *Oncidium, Arabidopsis, Oryza sativa, Glycine max,* and *Pisum sativum* was carried out. Among them, *O. sativa* shows high identity (82.1%) to *Oncidium* and contains GSH oxidation activity as *Oncidium* (data not shown). As shown in Figure 3A, the AsA-binding site is conserved in all *CytAPX1* proteins at Lys30Asn31Cys32Pro34Ile35His169Arg172 (black diamonds) (Sharp et al., 2003). After comparative analysis of the primary structure, 22 amino acid residues were found to vary between *Arabidopsis* and *Oncidium*, and also between *Arabidopsis* and *O. sativa* (as indicated by boxes and red background in Figure 3A). The conformation structural models of *G. max* and *P. sativum* were used as reference for further analysis of the three-dimensional conformation. The homology-derived structural model revealed that three amino acid residues in *OgCytAPX1* (Pro^63^, Asp^75^, and Tyr^97^) had different properties from the corresponding amino acids in AtCytAPX1 (Asp^63^, His^75^ and His^97^). In the structural model, these three amino acids are located at the surface, relatively close to heme and the AsA-binding site among the 22 amino acid residues (Figure 3B, colored red), and are proposed to be the key residues forming the GSH oxidation affinity (Figures 3B and 3C, colored cyan). Therefore these three amino acid residues in *OgCytAPX1* and corresponding residues in *AtCytAPX1* were chosen for site-directed mutagenesis assay to validate GPX activity. The following derived mutants in *OgCytAPX1* were thus generated: (1) single-residue mutation: Pro^63^Asp, Asp^75^His and Tyr^97^His, (2) double-residue mutation: Pro^63^Asp-Asp^75^His, Pro^63^Asp-Tyr^97^His and Asp^75^His-Tyr^97^His, and (3) triple-residues mutation: Pro^63^Asp-Asp^75^His- Tyr^97^His. Each mutant protein was expressed in *E. coli* BL21 cell, isolated, and purified for biochemical assay. Similar far-UV circular dichroism spectra for wild-type protein and mutants indicated their identical folds, which are not altered by amino acid replacement (Figure S1). Enzymatic activity assays demonstrated that all the mutated *OgCytAPX1* recombinant proteins, except the triple-residue mutation, retained both AsA and GSH oxidization activities (Figures 4A–4E). In a similar manner, the corresponding amino acid residues Asp^63^, His^75^, and His^97^ in *AtCytAPX1* were mutated to Pro63-Asp75-Tyr97 (as present in *OgCytAPX1*) and assays of AsA/GSH-oxidizing activity were carried out. Only the triple-residues mutation Asp63Pro-His75Asp-His97Tyr exhibited GPX activity (Figures 4B–4F). All the mutated CytAPX1 showed equal enzymatic activity of APX (Figures 4A–4D). These results demonstrated that Pro63Asp75Tyr97 are required for GSH oxidation activity of *OgCytAPX1*.

### Phylogeny of CytAPX1 Antioxidant Substrate Recognition Sites in Plants

**Figure 5:**
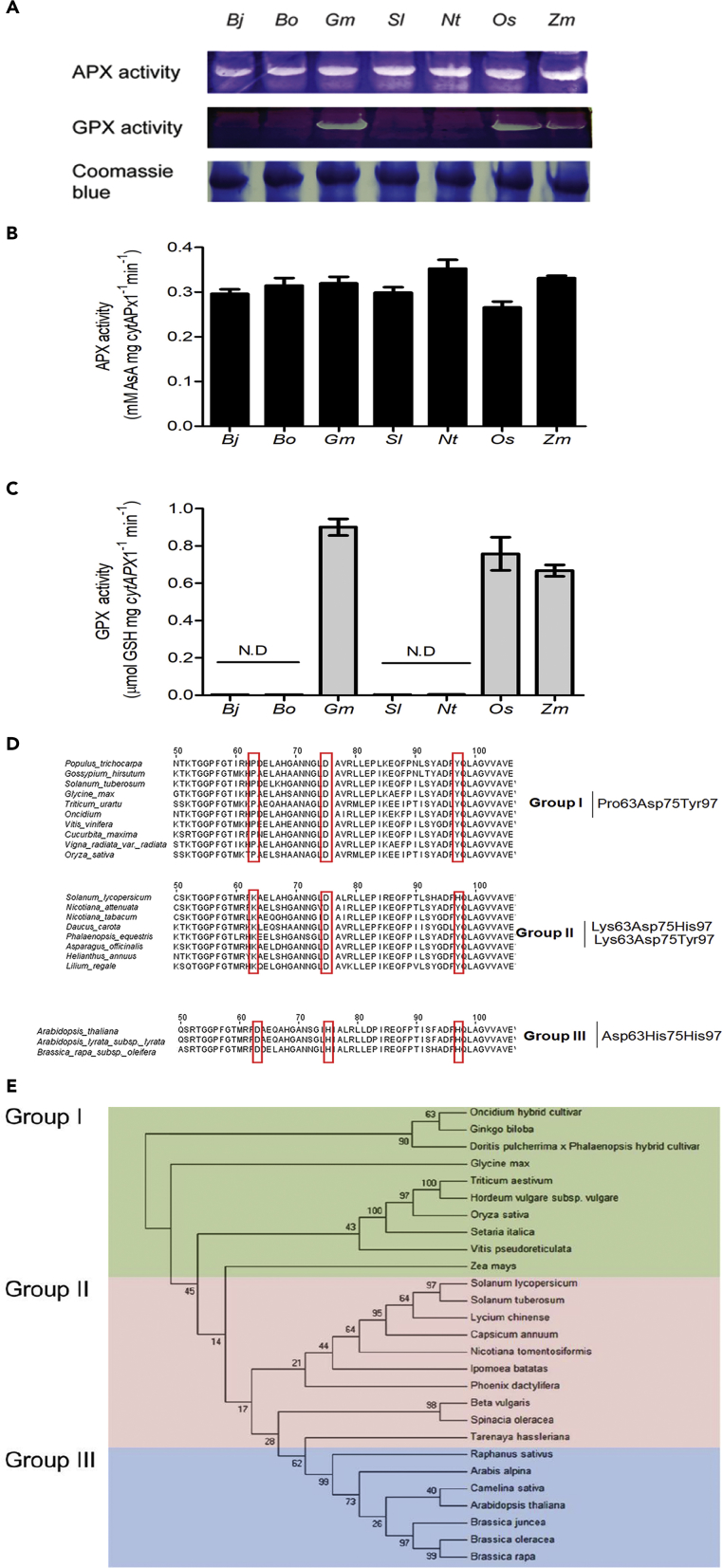
Biochemical Assays of APX and GPX Activities for Recombinant *CytAPX1* Proteins, Alignment of *CytAPX1s,* and the Phylogenetic Relationship of *CytAPX1s* From Various Plant Species (A) Recombinant proteins of CytAPX1 from various plants were expressed in *E. coli.* Each 50 μg purified recombinant protein was assayed *in-gel* for APX (upper gel) and GPX activities (middle gel). Coomassie blue staining was used as internal control (bottom gel). Recombinant proteins of *CytAPX1* were from *Bj*, *Brassica juncea; Bo*, *Brassica oleracea; Gm*, *Glycine max; Sl, Solanum lycopersicum; Nt*, *Nicotiana tabacum; Os*, *Oryza sativa;* and *Zm*, *Zea mays.* (B and C) APX and GPX activities of the 50 μg purified *CytAPX1* protein from various plants were measured by the associated histogram analysis, respectively. (D) Hypothetical classification of *Oncidium CytAPX1* and some related *CytAPX1s* from various plants based on the three key amino acid residues. Group I with typical type of Pro63Asp75Tyr97, Group II with transition type of Lys(Arg) (Ser) (Gln)63Asp(Asn) (Val) (Glu)75Tyr(His)97, and group III with atypical type of Asp(Glu)63His75His97. Red box highlighted the amino acid residues of Group I, II, and III classifications. (E) Phylogenetic analysis of *CytAPX1's* full-length amino acid sequence among 27 plant species. The phylogenetic tree was generated using the neighbor joining method with 1,000 bootstrap replications by MEGA6 software (Tamura et al., 2013). Green color highlights group I, pink color indicates group II, and blue color indicates group III.

To understand the universality of *CytAPX1* with dual substrate-binding specificity in planta, *CytAPX1* from seven plant species (*Brassica juncea*, *Brassica oleracea, O. sativa*, *Zea mays*, *G. max*, *Solanum lycopersicum*, and *Nicotiana tabacum*) were cloned and the recombinant proteins were produced from *E. coli* to assay for GPX activity in gel. Recombinant proteins from *G. max*, *O. sativa,* and *Z. mays* displayed both AsA and GSH oxidization activities, as *OgCytAPX1* did (Figures 5A–5C), whereas proteins from the other species did not. Analysis of the full amino acid sequences revealed that plant species are hypothetically classified into three groups based on amino acid composition of GSH-binding site. Group I, including *Oncidium*, *G. max*, *O. sativa,* and *Z. mays*, contains the typical residues Pro63Asp75Tyr97 referred to as the *Oncidium* type (Figure 5D). Group II, with one to two conserved amino acids to Group I, including *S. lycopersicum* and *N. tabacum*, contains residues Lys63Asp75His97 or Lys63Asp75Tyr97. Group III, with no conserved amino acids to Group I, including *Arabidopsis*, *B. juncea*, and *B. oleracea*, contains residues Asp63His75His97, referred to as the *Arabidopsis* type (Figure 5D). Only Group I plants, possessing the GSH oxidization activity conferred by Pro63Asp75Tyr97, exhibit dual substrate recognition for oxidizing both AsA and GSH, whereas plants of groups II and III do not (Figures 5A–5C). A phylogenetic relationship based on the full amino acid sequence of *CytAPX1* was constructed (Figure 5E). A total 27 plant species were grouped into three separate clades based on Maximum-Likelihood method. Group I comprises mainly eudicot Populus trichocapa, N. tabacum, G. max etc., but sequence similarities are more closely related to monocot Oncidium CytAPX1. On the other hand, 10 plant species of group II, including *S. lycopersicum* and *Nicotiana attenuata,* and 7 plant species of group III, including *Arabidopsis thaliana*, *Arabidopsis lyarata,* and *B. oleracea,* contain the atypical residues of Asp63His75His97 and do not exhibit GSH oxidization activity (Figures 5D and 5E). It is interesting to note that group II species have the transition-type residues between group I and group III. The clade marked with different colors denotes grouping classification of *CytAPX1s.* The result corresponds to group classification by three key amino acid residues (Figure 5E).

### Confirmation of GSH-Binding Activity in *OgCytAPX* by Isothermal Titration Calorimetry and UV-Vis Analysis

**Figure 6:**
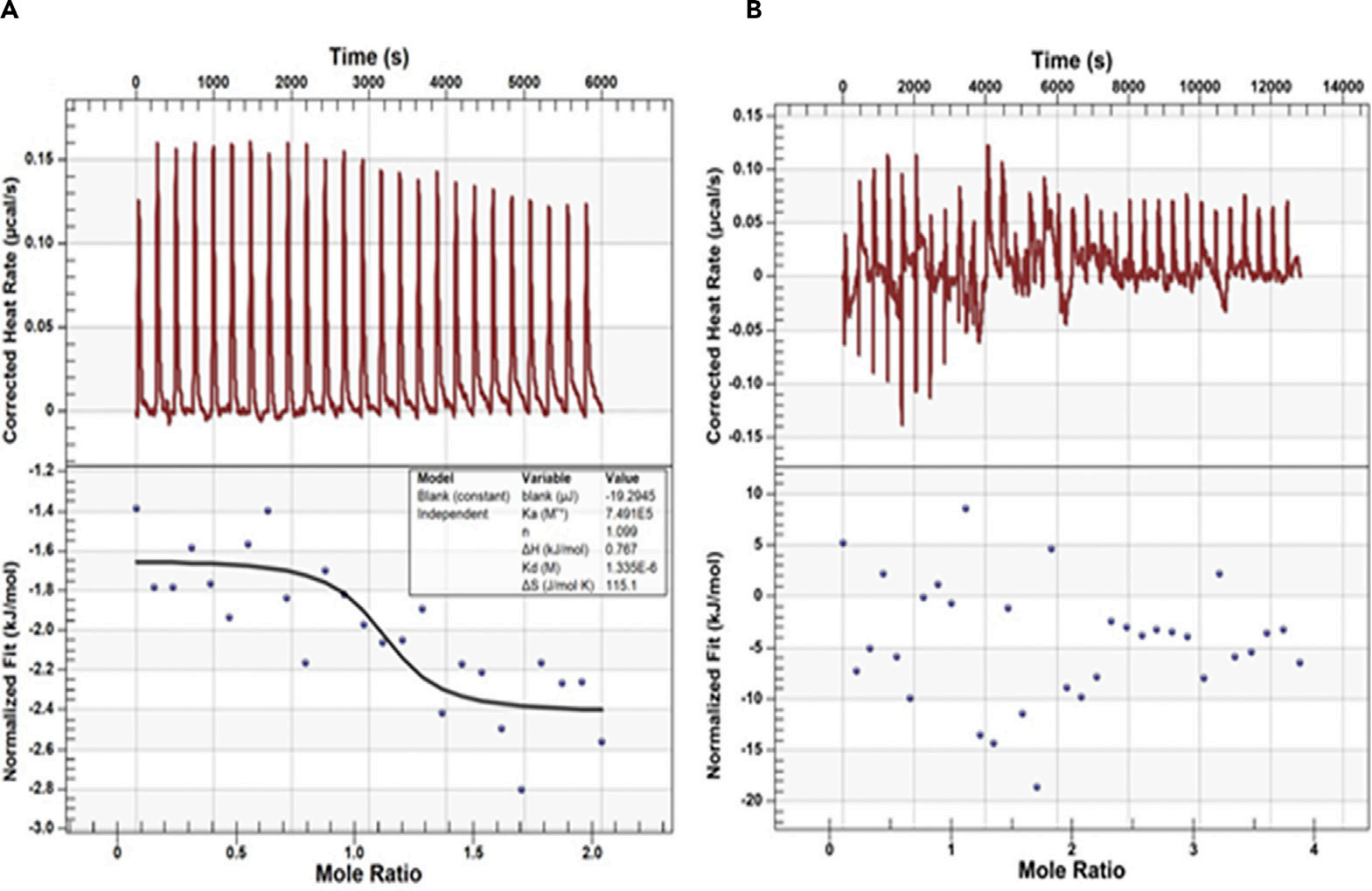
GSH Binding to Wild-type and Mutant Proteins of *OgCytAPX1* Isothermal titration calorimetry analysis of GSH binding to wild-type and mutant *OgCytAPX1*. Left panel, raw data in J/s versus time showing heat release on injection of (A) 2.0 mM GSH into a 980-L cell containing 0.1 mM wild-type *OgCytAPX1* and (B) 2.0 mM GSH into a 980-L cell containing 0.1 mM mutant *OgCytAPX1*. Right panel, integration of raw data yielding the heat per mole versus molar ratio. The inset shows thermodynamic parameters of each experiment.

**Figure 7:**
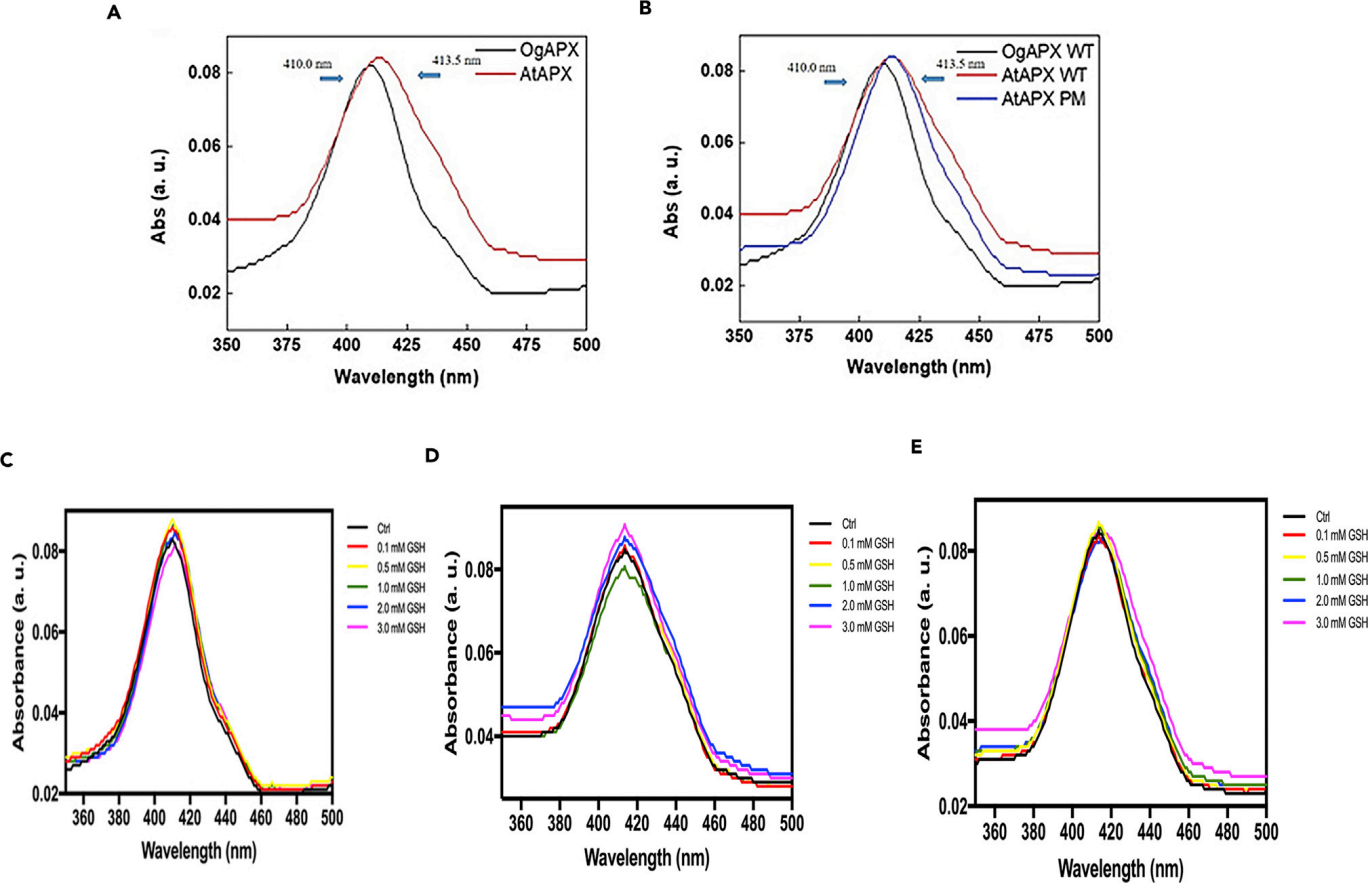
Optical Properties of *OgCytAPX1*-Heme and *AtCytAPX1*-Heme Complexes (A and B) UV-visible absorption spectra of (A) *OgCytAPX1* WT (black line) and *AtCytAPX1* WT (red line) and (B) *OgCytAPX1* WT (black line), *AtCytAPX1* WT (red line), and *AtCytAPX1* mutant (blue line) heme domains were monitored over 350–500 nm wavelength under room temperature. Proteins are at a concentration of ∼5 μM. Soret absorption maxima are at (A) 410.0 and 413.5 nm respectively, and (B) 410.0, 413.5, and 413.5 nm respectively. (C–E) Optical properties of *OgCytAPX1*-heme and *AtCytAPX1*-heme complexes with different initial concentrations of GSH. The Soret absorbtion maxima in (C) shifts from 410.0 nm to 411.5 nm, in (E) shifts from 413.5 to 415.0 nm, and in (D) shows no difference.

The GSH binding activity of *OgCytAPX1* was further validated by ITC analysis. As shown in Figure 6, GSH reacting with wild-type *OgCytAPX1* releases corrected heat rate (Figure 6A), whereas it does not happen in mutated *OgCytAPX1* (Figure 6B). The optical property assayed by UV-vis analysis revealed that Soret absorption maximal of *OgCytAPX1* and *AtCytAPX1* are around 410 nm (Figure 7A), and *AtCytAPX1* PM (mutation from Asp63His75His97 to Pro63 Asp75Tyr97) shows absorption spectra close to *OgCytAPX1* (Figure 7B). This demonstrated that *OgCytAPX1* is a heme-containing protein, same as *AtCytAPX1*.

The UV-vis analysis also provides strong evidence that Soret absorption maxima of *OgCytAPX1* is shifted from 410 to 411.5 nm (by +1.5 nm) in parallel with the titration concentration of GSH binding (Figure 7C). This suggests that heme group is oxidized during the GSH redox reaction. In contrast, absorption of *AtCytAPX1* is not affected by GSH titration (Figure 7D). However, the mutant of *AtCytAPX1* (PM) is shifted from 413.5 nm to 415 nm in parallel with GSH titration, as *OgCytAPX1* does (Figure 7E). This indicates that the substitution of Pro63, Asp75, and Tyr97 in CytAPX1 is critical for capability of GSH binding.

### Biochemical and Physiological Assays of Transgenic *Arabidopsis* Overexpressing *OgCytAPX1* and *AtCytAPX1,* Respectively

**Figure 8:**
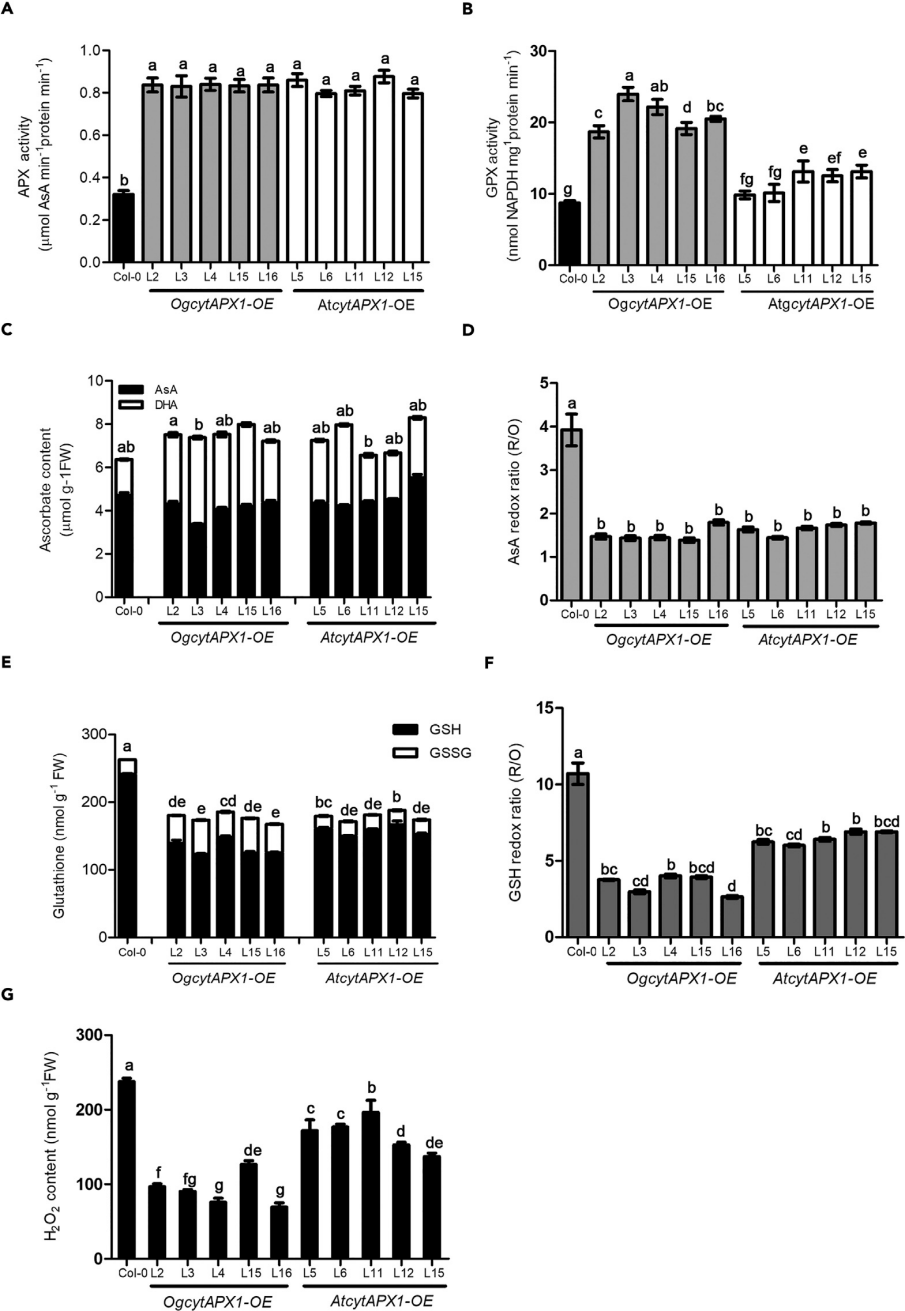
Effect of *Oncidium CytAPX1* and *Arabidopsis CytAPX1* on AsA and GSH Level/Redox Ratio after Overexpressing in *Arabidopsis* *CytAPX1* from *Oncidium* and *Arabidopsis* was overexpressed in *Arabidopsis*; five independent lines of each transformant plant were selected for monitoring the alternation of antioxidant levels and redox state. (A–G) (A) Similar APX activity shown in five selected independent lines of each gene transformant plant. Comparison of (B) GPX activity, (C) AsA (black bar) and dehydroascorbate (DHA) level (white bar), (D) AsA redox ratio, (E) GSH (black bar) and GSSG level (white bar), (F) GSH redox ratio, and (G) H_2_O_2_ levels, among *OgCytAPX1*-*OE*, *AtCytAPX1-OE,* and WT (Col-0). All the measurements except (A) and (B) were performed with plants grown at 30°C, 14 days, after transferring from 22°C. The activities of APX and GPX were measured from plants grown in 22°C, short day condition for 6 weeks. R/O: reduced form antioxidant to oxidized form antioxidant. Error bar indicates the SD (standard deviation of the mean n = 30). Statistical significance was analyzed by ANOVA with post-hoc test. Different letters indicate significant differences between wild-type and transgenic lines according to Fisher's protected least significant difference test at a significant level of p < 0.05.

*OgCytAPX1* exhibits substrate-oxidizing affinities for both AsA and GSH in H_2_O_2_ reduction, whereas *AtCytAPX1* exhibits only AsA-binding activity. To further examine their difference in function, *Arabidopsis* lines overexpressing either one of *OgCytAPX1* or *AtCytAPX1* were generated and APX activities in these lines were determined. Five independent lines for each gene transformation that had similar APX activity were selected for further study (Figure 8A). Transgenic *Arabidopsis* were grown for 6 weeks and transferred to high ambient temperature (30°C) for 14 days; then their GPX activity, AsA level and AsA redox ratio, GSH level, GSSG level and GSH redox ratio, as well as endogenous H_2_O_2_ content were measured. Higher total GPX activity was measured from *OgCytAPX1*-OE *Arabidopsis*, compared with Col-0 WT and *AtCytAPX1*-OE *Arabidopsis*, suggesting that *OgCytAPX1* potentially confers GPX activity in *Arabidopsis* (Figure 8B). Notably, while the transgenic *Arabidopsis* plants overexpressing either *OgCytAPX1* or *AtCytAPX1* were grown at ambient temperature (22°C), no significant differences in AsA and DHA level or in AsA redox ratio were observed (Figures 8C and 8D). This implied that *OgCytAPX1* and *AtCytAPX1* have equal abilities to oxidize AsA at ambient temperature. However, while they were grown at high ambient temperature (30°C), the endogenous GSH level (Figure 8E) and GSH redox ratio (Figure 8F) in *OgCytAPX1*-overexpressing *Arabidopsis* were significantly lower than those in *AtCytAPX1*-overexpressing *Arabidopsis*. This is in contrast to that of no difference growing in ambient temperature (22°C) (Figure S2). Moreover, the endogenous H_2_O_2_ content of transgenic *OgCytAPX1*-OE *Arabidopsis* plants was one-third lower than that of transgenic *AtCytAPX1*-OE *Arabidopsis* plants (Figure 8G), suggesting that the potential GPX activity in *OgCytAPX1* is effective to scavenge H_2_O_2_ and maintain the redox homeostasis at a lower H_2_O_2_ level, while plants stay at thermal stress condition, such as at 30°C condition.

**Figure 9:**
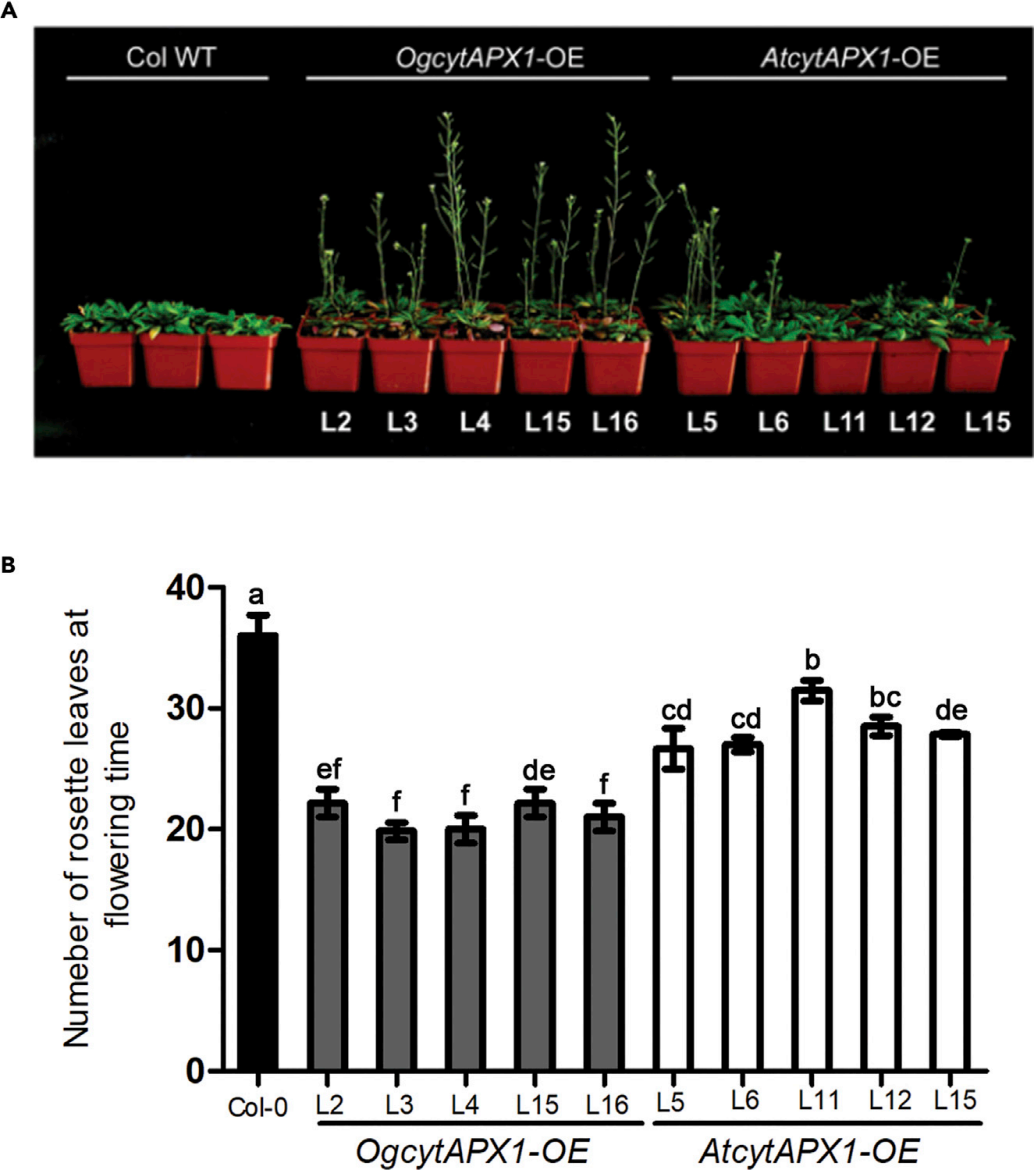
Effect of *Oncidium* CytAPX1 and *Arabidopsis* CytAPX1 on Flowering Time after Overexpressing in *Arabidopsis* *CytAPX1* from *Oncidium* and *Arabidopsis* was overexpressed in *Arabidopsis* to monitor the flowering time. (A) Photography shows *OgCytAPX1*-*OE* plants flowering earlier than *AtCytAPX1*-*OE* plants and WT. (B) Rosette leaves number of *OgCytAPX1*-*OE* plants, *AtCytAPX1*-*OE* plants, and WT while bolting. Experiments were repeated twice using 20 plants in each group. The number of rosette leaves was determined when inflorescences were 1 cm in length. Plants were first grown at 22°C under short day condition (8/16-h photoperiod) for 6 weeks and then transferred to 30°C under short day conditions until bolting. After bolting, plants were placed at 22°C under short day conditions for recovery from stress to determine the number of rosette leaves. Error bar indicates the SD (standard deviation of the mean [n = 40]). Statistical significance was analyzed by ANOVA with post-hoc test. Different letters indicate significant differences between wild-type and transgenic lines according to Fisher's protected least significant difference test at a significant level of p < 0.05.

Furthermore, observation of the flowering time revealed that all the transgenic *Arabidopsis* lines showed earlier flowering than wild-type (Col-0), once they were grown in high ambient temperature (30°C for 14 days). Moreover, *OgCytAPX1*-OE plants showed much earlier bolting than *AtCytAPX1*-OE plants (Figure 9A). The number of rosette leaves in *OgCytAPX1*-OE plants was approximately 20, compared with 25 leaves in *AtCytAPX1*-OE plants and 37 leaves in wild-type (Figure 9B). This implied that *OgCytAPX1* with dual antioxidant is beneficial to plants against environmental stress.

### *OgcytAPX1* Overexpression in *vtc1* Mutant Mitigates ROS Damage through the Direct Utilization of GSH and Enhances Tolerance to Heat and Salt Stress

**Figure 10:**
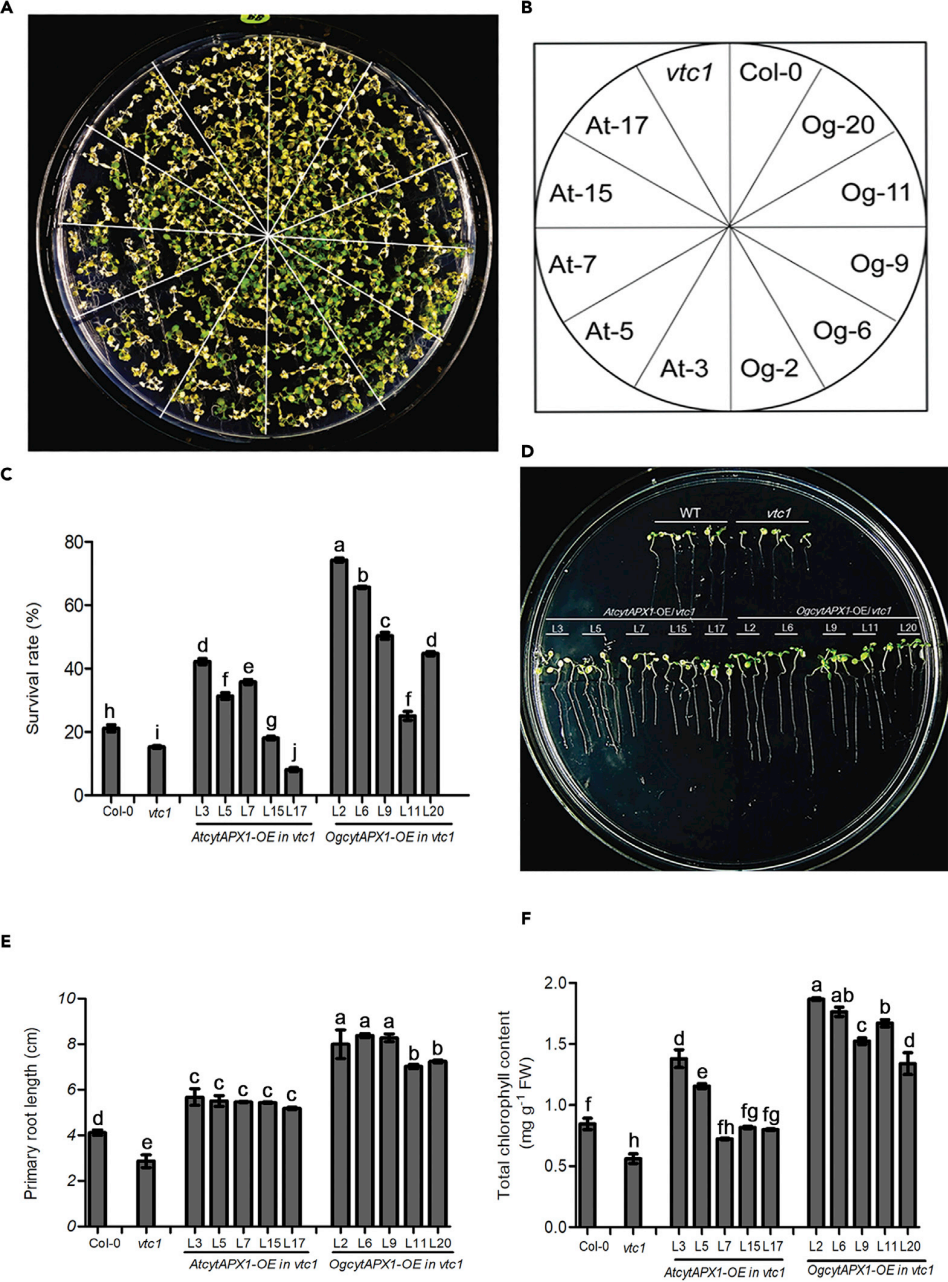
Effects of *Oncidium CytAPX1* and *Arabidopsis CytAPX1* on Heat and Salt Stress Tolerance after Overexpressing in *Arabidopsisvtc1*-Deficient Mutant (A and B) Phenotypic survival of *Arabidopsis* seedlings WT(Col-0), *vtc*1 mutant and overexpression lines of *OgCytAPX1-OE* and *AtCytAPX1-OE* after subjecting to heat stress at temperature 42° C for 2 hours in light/day photoperiod. (C–F) (C) Survival rate of transgenic *Arabidopsis* lines, *vtc1* mutant, and WT after treatment at 42°C for 2 h. Comparison of (D) salt stress tolerance, (E) the primary root length, (F) leaf chlorophyll content among *vtc1* mutant, WT (Col-0), and *OgcytAPX1*-OE and *AtcytAPX1-OE* in *vtc1* mutant after salt treatment with 150 mM NaCl for 2 weeks. Error bar indicates the SD (standard deviation of the mean [n = 30]). Statistical significance was analyzed by ANOVA with post-hoc test. Different letters indicates significant differences between wild-type and transgenic lines according to Fisher's protected least significant difference test at a significance level of p < 0.05.

*Arabidopsis vtc1* mutant, an ascorbate biosynthesis-deficient mutant, displays high sensitivity to environmental stresses. To examine the functional role of *OgcytAPX1* in enhancing stress tolerance under AsA starvation, *vtc1* mutants ectopically overexpressing *OgcytAPX1* and *AtcytAPX1* were produced. As shown in Figure 10, *OgcytAPX-OE-vtc1* displayed higher tolerance of 42°C heat stress for 2 h (Figures 10A–10C); the percentage of plants surviving stress treatment was 60% for *OgcytAPX-OE-vtc1*, compared with 20% for *AtcytAPX1-OE-vtc1*, 10% for *vtc1,* and 20% for wild-type *Arabidopsis*. The salt stress assay on 150 mM NaCl medium for 2 weeks exhibited that *OgcytAPX1* conferred elevated salt tolerance (Figure 10D). Root growth activity is much more vigorous in *OgcytAPX1-OE-vtc1* lines than in *AtcytAPX1-OE-vtc1* lines, *vtc1*, and Col-0 WT (Figure 10E). The total chlorophyll content also showed higher level than others (Figure 10F). Taken together, results clearly demonstrated that the function of the dual antioxidant-binding activities of *OgcytAPX1* is to enable plants to achieve a much higher tolerance of environmental stresses.

## Discussion

### Dual Antioxidant-Binding Specificities for AsA and GSH by *CytAPX1* Is an Evolutionary Event

APX is a heme peroxidase, found in all kingdoms of life, and typically catalyzes the one- and two-electron oxidation of a number of organic and inorganic substrates. Peroxidases of distinct families generally display representative sequence signatures and essential amino acids in heme cavity, and each family possesses a peculiar fold of the heme peroxidase domain (Zamocky et al., 2008). In the past decade, intensive analysis reveals that distinct families show pronounced catalase, cyclooxygenase, chlorite dismutase, or peroxygenase activities, in addition to the common peroxidatic activity (Zamocky et al., 2015). However, only ascorbate and cytochrome *c* peroxidases are typical monofunctional peroxidases with either ascorbate or cytochrome *c* as one-electron donor. The main function seems to be scavenging excess H_2_O_2_. Our present findings demonstrate that *OgCytAPX1* in *Oncidium* and some plants possesses an additional activity (or pathway) of GPX, besides APX activity, for H_2_O_2_ reduction. Enzyme kinetic analysis showed that the binding affinity of *OgCytAPX1* toward GSH is higher than toward AsA (Figures 2H and 2I). Maybe it is due to the lower concentration of GSH present in plant cell, about one-tenth of AsA (Noctor, 2006) (Table S1). The property of *OgCytAPX1* is distinct from that of most APXs in plants, such as *Arabidopsis* (Figure 2), *N. tabacum*, and *S. lycopersicum* (Figure 5). It suggests that a new phylogenetic clade of CytAPX1, which can use both AsA and GSH as electron donors, has been evolving.

Pro63, Asp75, and Tyr97 of OgCytAPX1 are required for GSH oxidation, and three groups of *CytAPX* in planta are classified based on the amino acid residues of GSH-binding site. Based on the structural conformation model and serial site-directed mutations, we concluded that the three residues, Pro63, Asp75, and Tyr97 are required for GSH oxidation activity (Figures 3 and 4). Whether there are any other amino acid residues involved in the formation of GSH-binding site, further investigation on structural function by using crystallographic approach is required. Plant species containing these three residues, Pro63, Asp75, and Tyr97, are designated as group I. In contrast, plants containing residues of Asp63His75His97 in the corresponding location and without GSH oxidation activity, such as in *Arabidopsis* and other plants of *Brassica* spp., are designated group III. The residue composition with either Lys63Asp75Tyr97 or Lys63Asp75His97 in group II is a transition type, such as *N. attenuata* and *S. tuberosum*, which have no GSH oxidation affinity either (Figure 5). Interestingly, it seems that more monocot plants belong to group I than eudicot plants (Figure 5). However, many active sites contain conserved substitution, which is structurally related to APX (Lazzarotto et al., 2011). Thus duplication event conserved in the chromosome region reflects that the active site varies for intragenomic duplication in *Oncidium*.

### The Function of *CytAPX* Is Associated with Redox Homeostasis and Flowering Induction under Environmental Stress

APX is one of the key enzymes involved in the regulation of ROS homeostasis during plant growth or development and under adverse stress conditions (Correa-Aragunde et al., 2013, Maruta et al., 2012, Suzuki et al., 2013). Our previous study has shown that *OgCytAPX1* is markedly upregulated to scavenge H_2_O_2_ by catalyzing AsA into DHA and causes a drastically reduced level of AsA and AsA redox ratio under high ambient temperature (30°C) (Chin et al., 2014). Also, high expression level with strong enzymatic activity of *CytAPX1* is associated with low GSH/high GSSG content and low GSH redox ratio under light, drought, salt stress (Faize et al., 2011, Hernández et al., 2000, Karpinski et al., 1997). In addition, the redox change of AsA, coupled with GSH redox state, plays a crucial role in protecting photosynthetic system from oxidative stress (Foyer and Noctor, 2011, Miller et al., 2010) and in floral induction in *Oncidium* (Chin et al., 2016). These reports support that *CytAPX1* together with DHAR is the main enzyme regulating the redox homeostasis in AsA-GSH cycle (Gallie, 2013, Le Martret et al., 2011). The present study demonstrated that *OgCytAPX1* possesses two substrate oxidation specificities for AsA and GSH, and has additional GPX activity (Figure 2). OgCytAPX1 causing lower H_2_O_2_ level and lower GSH redox ratio than *AtCytAPX1* was demonstrated in overexpressing *Arabidopsis* (Figure 8). Our work strongly supports that GSH consumption by *OgCytAPX1* is an independent biochemical step of AsA-GSH cycle.

Alternation of GSH level and GSH redox ratio affecting flowering time in *Arabidopsis*, wheat, and *Eustoma grandiflorum* have been reported (Yanagida et al., 2004) (Gulyas et al., 2014, Hatano-Iwasaki and Ogawa, 2012). Also, GSH level or redox status mediating GSH redox potential changes and glutathionylation from oxidative stress was known to associate with redox signal transduction to affect growth and development in plants (Noctor et al., 2012, Shigeoka and Maruta, 2014). The earlier flowering in association with lower GSH redox ratio in *OgCytAPX1*-OE *Arabidopsis* strongly suggests that the dual substrate recognition function of *OgCytAPX1* is more efficient to scavenge H_2_O_2,_ regulate redox homeostasis, and trigger signal transduction to affect development under environmental changes (Figure 9).

### *CytAPX1* Uses GSH to Enhance the Capability in Scavenging ROS and Confers High Tolerance on Plants in Oxidative Stress

Furthermore, understanding the truly physiological function of dual antioxidant recognition in *OgcytAPX1* is the issue of most concern in this work. We used *Arabidopsis vtc1*mutant, which is an ascorbate biosynthesis-deficient mutant, lacking ascorbate for ROS detoxification (Smirnoff, 2000), and displays high sensitivity to environmental stresses, such as high temperature and salinity (Wang et al., 2003, Larkindale et al., 2005). Obviously, *OgcytAPX1* conferred greater tolerance and survival in transgenic *vtc1* plants compared with *AtcytAPX1* under heat and salt stress (Figure 10). The similar pattern of AsA level and redox ratio compared with *AtcytAPX1*-*OE*-*vtc1 Arabidopsis* suggests that the greater tolerance of *OgcytAPX1-OE vtc1 Arabidopsis* is not due to the effect of AsA oxidation to scavenge H_2_O_2_, but rather critical is the outcome of GSH consumption. The data demonstrate that the physiological function of *OgcytAPX1* is able to compensate for AsA deficiency to mitigate ROS oxidative damage in AsA-deficient *vtc1* system. While plants are under environmental stress, AsA is enormously employed for ROS scavenging and is associated with several phytohormones and signaling compound biosynthesis, such as abscisic acid, nitric oxide (NO), ethylene, and salicylic acid (Bethke et al., 2004, Khan et al., 2011, Kerchev et al., 2011). Consequently, AsA pool size decreases markedly in light, drought, heat stress, and high ambient temperature (Bartoli et al., 2005, Song et al., 2005, Chin et al., 2014). Thus it suggests that the capability of *OgcytAPX1* to use GSH under AsA starvation condition confers high tolerance on plants in unfavorable environmental conditions.

**Figure 11:**
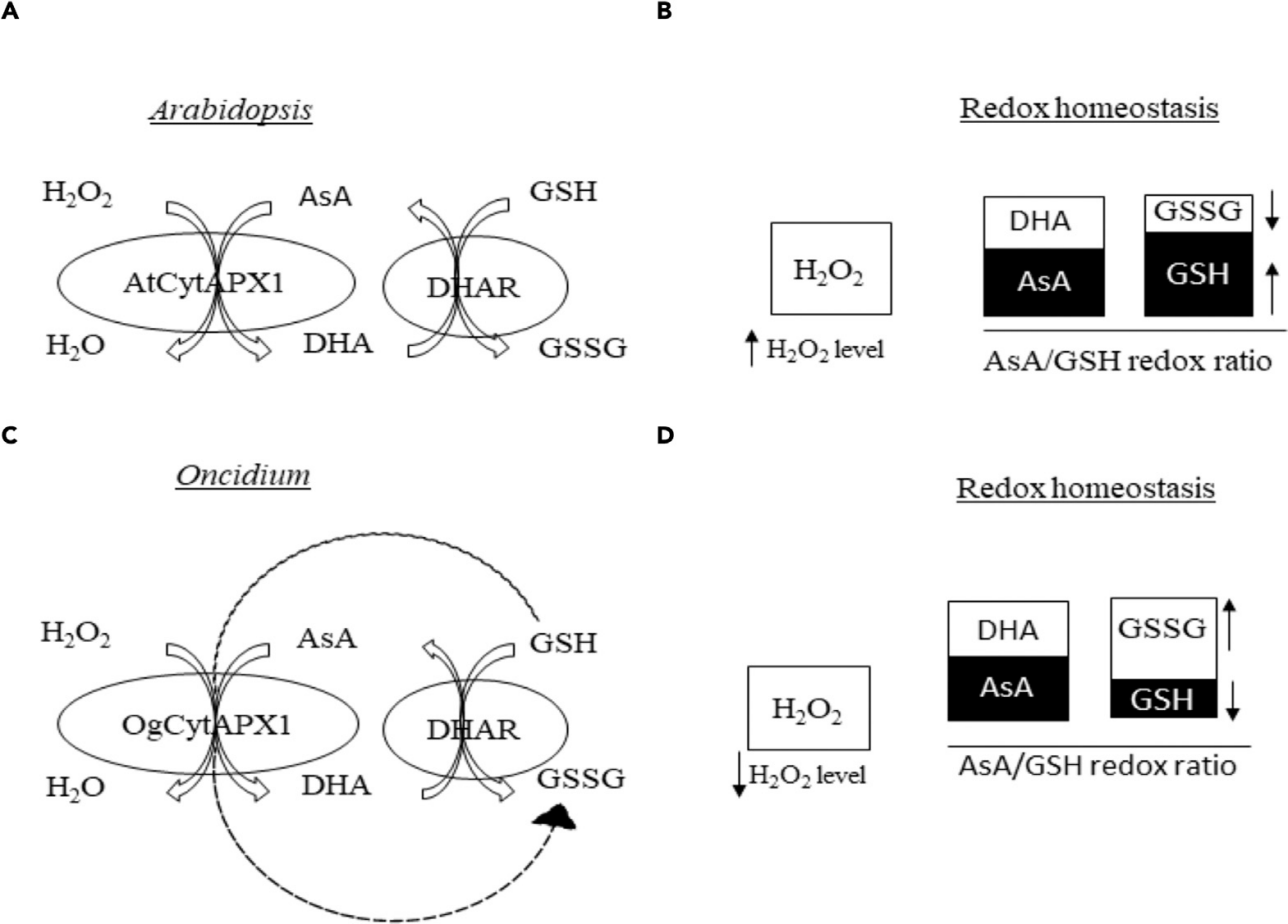
Schematic Representation of the General and Variant Form of AsA-GSH Cycle Existing in *Arabidopsis* and *Oncidium* (A and B) The general form of AsA-GSH system shows higher H_2_O_2_ level, higher level of DHA/AsA ratio, and higher GSH/GSSG ratio under stress, suggesting low efficiency of ROS homeostasis regulation. (C and D) The variant form of AsA and GSH redox system found in *Oncidium* and several plants shows lower H_2_O_2_ level, lower level of DHA/AsA ratio, and lower GSH/GSSG ratio under stress. The effective antioxidative regulatory system is due to the additional function of glutathione consumption in *OgAPX1* (shown by dashed line).

In summary, we have discovered that *CytAPX* of *Oncidium* orchid and of several plants has an additional GSH oxidization activity to facilitate redox homeostasis under high temperature stress condition (Figure 11). This is coincident with the strong growth potential of *Oncidium* and several monocot plants in adaption to wild field. The three amino acid residues Pro63, Asp75, and Tyr97, required for GSH oxidization, were identified. Our study pointed out that the GSH oxidation activity in *CytAPX* distributes in most monocot plants, such as *O. sativa, Z. mays*, and *G. max*. Phylogenetic relationship based on the variation of GSH-binding residues of *CytAPX* showed that the phylogenetic clade is classified into three groups. The significance and evolutionary mechanism in plants are worthy of further investigation.

### Limitations of Study

This work first describes that OgcytAPX1 possesses two substrate oxidation specificities for AsA and GSH and has the additional GPX activity to facilitate redox homeostasis under high temperature stress conditions. We confirmed that OgcytAPX1 causing lower H_2_O_2_ level and lower GSH redox ratio than *AtcytAPX1* in *Arabidopsis* overexpression lines suggested that OgcytAPX1 uses GSH as an independent biochemical step in AsA-GSH cycle. UV-vis analysis further confirmed its heme-containing protein. Furthermore, we discovered three amino acid residues Pro63, Asp75, and Tyr97, required for GSH oxidation. Our investigation pointed out that the GSH oxidization activity in cytAPX1 distributes in many plants, such as *O. sativa, Z. mays*, and *G. max*, signifying the evolutionary mechanism in plants.

## Methods

All methods can be found in the accompanying Transparent Methods supplemental file.

## Supplemental Information

Document S1. Transparent Methods, Figures S1 and S2, and Table S1

## References

[bib54] Bartoli C.G., Guiamet J.J., Kiddle G., Pastori G.M., Di Cagno R., Theodoulou F.L. and Foyer C.H., Ascorbate content of wheat leaves is not determined by maximal L-galactono-1,4-lactono dehydrogenase (GalLDH) activity under drought stress. Plant Cell Environ. 28, 2005, 1073-1081.

[bib1] Barton, G.J.. (1993). ALSCRIPT: a tool to format multiple sequence alignments. Protein Eng. 6, 37-40. 10.1093/protein/6.1.37 8433969

[bib2] Bashandy, T., Guilleminot, J., Vernoux, T., Caparros-Ruiz, D., Ljung, K., Meyer, Y., and Reichheld, J.P.. (2010). Interplay between the NADP-linked thioredoxin and glutathione systems in Arabidopsis auxin signaling. Plant Cell 22, 376-391. 10.1105/tpc.109.071225 PMC2845418 20164444

[bib55] Bethke P.C., Badger M.R. and Jones R.L., Apoplastic synthesis of nitric oxide by plant tissues, Plant Cell 16, 2004, 332-341 10.1105/tpc.017822 PMC341907 14742874

[bib4] Chin, D.C., Hsieh, C.C., Lin, H.Y., and Yeh, K.W.. (2016). A low glutathione redox state couples with a decreased ascorbate redox ratio to accelerate flowering in oncidium orchid. Plant Cell Physiol. 57, 423-436. 10.1093/pcp/pcv206 26738548

[bib5] Chin, D.C., Shen, C.H., SenthilKumar, R., and Yeh, K.W.. (2014). Prolonged exposure to elevated temperature induces floral transition via up-regulation of cytosolic ascorbate peroxidase 1 and subsequent reduction of the ascorbate redox ratio in Oncidium hybrid orchid. Plant Cell Physiol. 55, 2164-2176. 10.1093/pcp/pcu146 25320212

[bib6] Correa-Aragunde, N., Foresi, N., Delledonne, M., and Lamattina, L.. (2013). Auxin induces redox regulation of ascorbate peroxidase 1 activity by S-nitrosylation/denitrosylation balance resulting in changes of root growth pattern in Arabidopsis. J. Exp. Bot. 64, 3339-3349. 10.1093/jxb/ert172 23918967

[bib8] De Pinto, M.C., Locato, V., and De Gara, L.. (2012). Redox regulation in plant programmed cell death. Plant Cell Environ. 35, 234-244. 10.1111/j.1365-3040.2011.02387.x 21711357

[bib10] Faize, M., Burgos, L., Faize, L., Piqueras, A., Nicolas, E., Barba-Espin, G., Clemente-Moreno, M.J., Alcobendas, R., Artlip, T., and Hernandez, J.A.. (2011). Involvement of cytosolic ascorbate peroxidase and Cu/Zn-superoxide dismutase for improved tolerance against drought stress. J. Exp. Bot. 62, 2599-2613. 10.1093/jxb/erq432 21239380

[bib11] Foyer, C.H., and Noctor, G.. (2011). Ascorbate and glutathione: the heart of the redox hub. Plant Physiol. 155, 2-18. 10.1104/pp.110.167569 PMC3075780 21205630

[bib12] Foyer, C.H., and Noctor, G.. (2016). Stress-triggered redox signalling: what's in pROSpect? Plant Cell Environ. 39, 951-964. 10.1111/pce.12621 26264148

[bib13] Foyer, C.H., and Shigeoka, S.. (2011). Understanding oxidative stress and antioxidant functions to enhance photosynthesis. Plant Physiol. 155, 93-100. 10.1104/pp.110.166181 PMC3075779 21045124

[bib14] Frendo, P., Baldacci-Cresp, F., Benyamina, S.M., and Puppo, A.. (2013). Glutathione and plant response to the biotic environment. Free Radic. Biol. Med. 65, 724-730. 10.1016/j.freeradbiomed.2013.07.035 23912161

[bib15] Gallie, D.R.. (2013). The role of L-ascorbic acid recycling in responding to environmental stress and in promoting plant growth. J. Exp. Bot. 64, 433-443. 10.1093/jxb/ers330 23162122

[bib16] Gest, N., Gautier, H., and Stevens, R.. (2013). Ascorbate as seen through plant evolution: the rise of a successful molecule? J. Exp. Bot. 64, 33-53. 10.1093/jxb/ers297 23109712

[bib18] Gulyas, Z., Boldizsar, A., Novak, A., Szalai, G., Pal, M., Galiba, G., and Kocsy, G.. (2014). Central role of the flowering repressor ZCCT2 in the redox control of freezing tolerance and the initial development of flower primordia in wheat. BMC Plant Biol. 14, 91. 10.1186/1471-2229-14-91 PMC4021066 24708599

[bib19] Hatano-Iwasaki, A., and Ogawa, K.. (2012). Overexpression of GSH1 gene mimics transcriptional response to low temperature during seed vernalization treatment of Arabidopsis. Plant Cell Physiol. 53, 1195-1203. 10.1093/pcp/pcs075 22628560

[bib20] Hernandez, J.A., Jimenez, A., Mullineaux, P., and Sevilia, F.. (2000). Tolerance of pea (Pisum sativum L.) to long-term salt stress is associated with induction of antioxidant defences. Plant Cell Environ. 23, 853-862.

[bib21] Karpinski, S., Escobar, C., Karpinska, B., Creissen, G., and Mullineaux, P.M.. (1997). Photosynthetic electron transport regulates the expression of cytosolic ascorbate peroxidase genes in Arabidopsis during excess light stress. Plant Cell 9, 627-640. 10.1105/tpc.9.4.627 PMC156944 9144965

[bib56] Kerchev P. I., Pellny T. K., Vivancos P. D., Kiddle G., Hedden P., Driscoll S., Vanacker H., Verrier P., Hancook R.D., Fover C.H., The transcription factor ABI4 is required for the ascorbic acid-dependent regulation of growth and regulation of jasmonate-dependent defense signaling pathways in Arabidopsis, Plant Cell 23, 2011, 3319-3334. 10.1105/tpc.111.090100 PMC3203439 21926335

[bib57] Khan T. A., Mazid M. and Mohammad, F., A review of ascorbic acid potentialities against oxidative stress induced in plants, J. Agrobiol. 28, 2011, 97-111

[bib22] Kotchoni, S.O., Larrimore, K.E., Mukherjee, M., Kempinski, C.F., and Barth, C.. (2009). Alterations in the endogenous ascorbic acid content affect flowering time in Arabidopsis. Plant Physiol. 149, 803-815. 10.1104/pp.108.132324 PMC2633856 19028878

[bib23] Koussevitzky, S., Suzuki, N., Huntington, S., Armijo, L., Sha, W., Cortes, D., Shulaev, V., and Mittler, R.. (2008). Ascorbate peroxidase 1 plays a key role in the response of Arabidopsis thaliana to stress combination. J. Biol. Chem. 283, 34197-34203. 10.1074/jbc.M806337200 PMC2590703 18852264

[bib24] Larkin, M.A., Blackshields, G., Brown, N.P., Chenna, R., McGettigan, P.A., McWilliam, H., Valentin, F., Wallace, I.M., Wilm, A., Lopez, R., et al. (2007). Clustal W and clustal X version 2.0. Bioinformatics 23, 2947-2948. 10.1093/bioinformatics/btm404 17846036

[bib58] Larkindale J., Hall J.D., Knight M.R. and Vierling E., Heat stress phenotypes of Arabidopsis mutants implicate multiple signaling pathways in the acquisition of thermotolerance, Plant Physiol. 138, 2005, 882–897 10.1104/pp.105.062257 PMC1150405 15923322

[bib25] Lazzarotto, F., Teixeira, F.K., Rosa, S.B., Dunand, C., Fernandes, C.L., Fontenele Ade, V., Silveira, J.A., Verli, H., Margis, R., and Margis-Pinheiro, M.. (2011). Ascorbate peroxidase-related (APx-R) is a new heme-containing protein functionally associated with ascorbate peroxidase but evolutionarily divergent. New Phytol. 191, 234-250. 10.1111/j.1469-8137.2011.03659.x 21352234

[bib26] Le Martret, B., Poage, M., Shiel, K., Nugent, G.D., and Dix, P.J.. (2011). Tobacco chloroplast transformants expressing genes encoding dehydroascorbate reductase, glutathione reductase, and glutathione-S-transferase, exhibit altered anti-oxidant metabolism and improved abiotic stress tolerance. Plant Biotechnol. J. 9, 661-673. 10.1111/j.1467-7652.2011.00611.x 21450042

[bib28] Maruta, T., Inoue, T., Noshi, M., Tamoi, M., Yabuta, Y., Yoshimura, K., Ishikawa, T., and Shigeoka, S.. (2012). Cytosolic ascorbate peroxidase 1 protects organelles against oxidative stress by wounding- and jasmonate-induced H(2)O(2) in Arabidopsis plants. Biochim. Biophys. Acta 1820, 1901-1907. 10.1016/j.bbagen.2012.08.003 22921811

[bib29] Miller, G., Suzuki, N., Ciftci-Yilmaz, S., and Mittler, R.. (2010). Reactive oxygen species homeostasis and signalling during drought and salinity stresses. Plant Cell Environ. 33, 453-467. 10.1111/j.1365-3040.2009.02041.x 19712065

[bib34] Noctor, G.. (2006). Metabolic signalling in defence and stress: the central roles of soluble redox couples. Plant Cell Environ. 29, 409-425. 10.1111/j.1365-3040.2005.01476.x 17080595

[bib35] Noctor, G., Mhamdi, A., Chaouch, S., Han, Y., Neukermans, J., Marquez-Garcia, B., Queval, G., and Foyer, C.H.. (2012). Glutathione in plants: an integrated overview. Plant Cell Environ. 35, 454-484. 10.1111/j.1365-3040.2011.02400.x 21777251

[bib36] Ogawa, K.. (2005). Glutathione-associated regulation of plant growth and stress responses. Antioxid. Redox Signal. 7, 973-981. 10.1089/ars.2005.7.973 15998252

[bib37] Pandey, S., Fartyal, D., Agarwal, A., Shukla, T., James, D., Kaul, T., Negi, Y.K., Arora, S., and Reddy, M.K.. (2017). Abiotic stress tolerance in plants: myriad roles of ascorbate peroxidase. Front. Plant Sci. 8, 581. 10.3389/fpls.2017.00581 PMC5397514 28473838

[bib38] Pignocchi, C., and Foyer, C.H.. (2003). Apoplastic ascorbate metabolism and its role in the regulation of cell signalling. Curr. Opin. Plant Biol. 6, 379-389. 10.1016/s1369-5266(03)00069-4 12873534

[bib41] Rosa, S.B., Caverzan, A., Teixeira, F.K., Lazzarotto, F., Silveira, J.A., Ferreira-Silva, S.L., Abreu-Neto, J., Margis, R., and Margis-Pinheiro, M.. (2010). Cytosolic APx knockdown indicates an ambiguous redox responses in rice. Phytochemistry 71, 548-558. 10.1016/j.phytochem.2010.01.003 20129631

[bib60] Song X.S., Hu W.H., Mao W.H., Ogweno J.O., Zhou Y.H. and Yu J.Q., Response of ascorbate peroxidase isoenzymes and ascorbate regeneration system to abiotic stresses in Cucumis sativus L., Plant Physiol Biochem 43, 2005, 1082-1088 10.1016/j.plaphy.2005.11.003 16386429

[bib43] Sharma, P., Jha, A.B., Dubey, R.S., and Pessarakli, M.. (2012). Reactive oxygen species, oxidative damage, and antioxidative defense mechanism in plants under stressful conditions. J. Bot. 2012, 26.

[bib44] Sharp, K.H., Mewies, M., Moody, P.C., and Raven, E.L.. (2003). Crystal structure of the ascorbate peroxidase-ascorbate complex. Nat. Struct. Biol. 10, 303-307. 10.1038/nsb913 12640445

[bib45] Shigeoka, S., and Maruta, T.. (2014). Cellular redox regulation, signaling, and stress response in plants. Biosci. Biotechnol. Biochem. 78, 1457-1470. 10.1080/09168451.2014.942254 25209493

[bib46] Suzuki, N., Koussevitzky, S., Mittler, R., and Miller, G.. (2012). ROS and redox signalling in the response of plants to abiotic stress. Plant Cell Environ. 35, 259-270. 10.1111/j.1365-3040.2011.02336.x 21486305

[bib47] Suzuki, N., Miller, G., Sejima, H., Harper, J., and Mittler, R.. (2013). Enhanced seed production under prolonged heat stress conditions in Arabidopsis thaliana plants deficient in cytosolic ascorbate peroxidase 2. J. Exp. Bot. 64, 253-263. 10.1093/jxb/ers335 PMC3528037 23183257

[bib48] Tamura, K., Stecher, G., Peterson, D., Filipski, A., and Kumar, S.. (2013). MEGA6: molecular evolutionary genetics analysis version 6.0. Mol. Biol. Evol. 30, 2725-2729. 10.1093/molbev/mst197 PMC3840312 24132122

[bib59] Wang W., Vinocur B. and Altman A., Plant responses to drought, salinity and extreme temperatures: towards genetic engineering for stress tolerance, Planta 218, 2003, 1-14. 10.1007/s00425-003-1105-5 14513379

[bib50] Yanagida, M., Mino, M., Iwabuchi, M., and Ogawa, K.. (2004). Reduced glutathione is a novel regulator of vernalization-induced bolting in the rosette plant Eustoma grandiflorum. Plant Cell Physiol. 45, 129-137. 10.1093/pcp/pch030 14988483

[bib51] Yu, X., Pasternak, T., Eiblmeier, M., Ditengou, F., Kochersperger, P., Sun, J., Wang, H., Rennenberg, H., Teale, W., Paponov, I., et al. (2013). Plastid-localized glutathione reductase2-regulated glutathione redox status is essential for Arabidopsis root apical meristem maintenance. Plant Cell 25, 4451-4468. 10.1105/tpc.113.117028 PMC3875729 24249834

[bib52] Zamocky, M., Hofbauer, S., Schaffner, I., Gasselhuber, B., Nicolussi, A., Soudi, M., Pirker, K.F., Furtmuller, P.G., and Obinger, C.. (2015). Independent evolution of four heme peroxidase superfamilies. Arch. Biochem. Biophys. 574, 108-119. 10.1016/j.abb.2014.12.025 PMC4420034 25575902

[bib53] Zamocky, M., Jakopitsch, C., Furtmuller, P.G., Dunand, C., and Obinger, C.. (2008). The peroxidase-cyclooxygenase superfamily: reconstructed evolution of critical enzymes of the innate immune system. Proteins 72, 589-605. 10.1002/prot.21950 18247411

