# Supplementary material for: Plant Cytosolic Ascorbate Peroxidase with Dual Catalytic Activity Modulates Abiotic Stress Tolerances
Source: iScience. 2019 May 15;16:31–49. doi: 10.1016/j.isci.2019.05.014 (PMC6542772; doi:10.1016/j.isci.2019.05.014)
Supplement: Document S1. Transparent Methods, Figures S1 and S2, and Table S1 [file mmc1.pdf]

ISCI, Volume *16*

Supplemental Information

Plant Cytosolic Ascorbate Peroxidase with Dual Catalytic Activity Modulates Abiotic Stress Tolerances

Dan-Chu Chin, Rajendran Senthil Kumar, Ching-Shu Suen, Chia-Yu Chien, Ming-Jing Hwang, Chun-Hua Hsu, Xu Xuhan, Zhong Xiong Lai, and Kai-Wun Yeh

Supplemental information

Transparent Methods

Plant materials and growth condition

Oncidium ‘Grower Ramsey’ plants were grown and maintained in a green house at 20-25°C for the observation of normal growth, following protocol described previously (Chin et al., 2016). For various biochemical assays, orchid plants were shifted and grown in phytotron at 30°C with 16-h-light/8-h-dark cycle photoperiod for the observation of number of inflorescence bud developed (Chin et al., 2016).For all biochemical activity assay, crude soluble proteins were obtained from *Oncidium* pseduobulb and *Arabidopsis* tissues. The tissues were ground to fine powder in liquid nitrogen and then homogenized with the extraction buffer containing 50mM sodium phosphate buffer, pH 7.2, 2mM EDTA,2mM DTT, 20% glycerol and PVPP, following protocol described previously (Chin et al., 2016).Transgenic *Arabidopsis* from the Col-0 SALK-line *vtc1* mutants and ectopically overexpressing *Oncidium* genes (*OgCytAPx1*) and *Arabidopsis* gene (*AtCytAPX1*), were grown at 22°C under short-day (SD) conditions (8 /16 hr photoperiod) for 6 weeks and subsequently maintained at 22°Cor transferred to 30°C to conduct various biochemical assays and to determine the number of leaves prior to floral initiation. *Oncidium* orchid used RNA extraction was grown on peat moss in pot at 22-30°C under a 16-h-light/8-h-dark cycle.

Gene cloning by RACE

Sequences of the full-length *CytAPX1s* from various plant species were completed using the rapid amplification of cDNA ends (RACE) method (GeneRacer RLM-RACE kit, Invitrogen™). Sequence was identified, confirmed and deposited in GeneBank under the assigned accession numbers.

Recombinant protein expression and purification

Recombinant protein expression and purification were performed by the pMAL™ Protein Fusion and Purification System (New England BioLabs). cDNAs for the target proteins were cloned into the pMAL-c5X vector and were transformed into *E. coli* (BL21-Codon-Plus). The recombinant proteins expressed in *E. coli* following 37°C incubation for 2 h (OD_{600} ~0.5), and induction with 100 μM isopropyl β-thiogalactopyranoside (IPTG) for 4 h at 28 °C. Harvested cells were sonicated with 10-s pulses separated by 10-s intervals for 10 min, and the insoluble materials were removed by centrifugation at 16,000x g for 15 min at 4 °C. The supernatant was sterile-filtered and the fusion protein was purified on a 2.5 x 10 cm column with amylose resin (New England BioLabs). The purified target protein fused with MBP (maltose binding protein) tag was digested by Factor-Xa (New England BioLabs) to remove the tag, and the digested proteins were monitored by 10% SDS-PAGE.

In-gel assay for ascorbate peroxidase (APX) and glutathione peroxidase (GPX) activity

The *in-gel* assay for APX activity was carried out following the method described previously (Mittler and Zilinskas, 1993). Appropriate amount of homogenates and the purified recombinant proteins were resolved using 10% native gel electrophoresis. Following electrophoresis, the gel was equilibrated with 50 mM sodium phosphate buffer (pH 7.0) and 2 mM ascorbate for a total of 30 min with the equilibration buffer changed every 10 min. The gel was then incubated with 50 mM sodium phosphate buffer (pH 7.0) containing 4 mM ascorbate and 20 mM H_2O_2 for 20 min. The gel was subsequently

washed with sodium phosphate buffer (pH 7.8), 28 mM tetramethylethylenediamine (TEMED) and 24 mM Nitro Blue Tetrazolium (NBT) with gentle agitation for approximately 10 min and the reaction stopped by a brief wash with distilled water. After staining, an achromatic band appeared against the dark purple background.

The *in-gel* assay for GPX activity was performed following the method described previously(Lin et al., 2002). Appropriate amount of homogenates and purified recombinant proteins were resolved using 10% native gel electrophoresis. After native PAGE, gel was submerged for 20 min twice in 50 mM Tris-HCl buffer (pH 7.9) before activity staining. Activity staining of GPX was as follows (MP method). Gel was soaked in the substrate solution (50 mM Tris-HCl buffer, pH 7.9, 13 mM GSH, and 0.004% hydrogen peroxide) with gentle shaking for 10–20 min. After a brief rinse, the GPX activity was developed in darkness at room temperature with 1.2 mM 3-(4,5-dimethylthiazol-2-yl)-2,5-diphenyl tetrazolium bromide (MTT) and 1.6 mM phenazine methosulfate (PMS) in distilled water for 10 min. The clear zone of GPX activity is present against a purple background.

APX and GPX activity assay

Ascorbate peroxidase was assayed by the method described previously (Nakano and Asada, 1981). The reaction mixture to measure APX activity contained 50 mM sodium phosphate buffer (pH 7.0), 0.2 mM EDTA, 0.5 mM AsA, 2 mM H_2O_2 (all the component are from Sigma) and 2mL of crude protein extract in a total volume 5mL. The activity was recorded as decrease in absorbance at OD_{290} for 1 min and the amount of ascorbate oxidized was calculated from the extinction coefficient 2.6 mM^{-1} cm^{-1}.

Activity of GPX from the transgenic *Arabidopsis* was assayed by a modified method

of the coupled enzyme system (Drotar et al., 1985). The reaction solution includes 50mM potassium phosphate (pH 7.0), 2mM H_2O_2, 2mM GSH, 2.5 units of glutathione reductase, 2mM 0.1 mM NADPH (all the component are from Sigma), and 2mL of desalted crude protein extract in a total volume 5mL. The reaction rate was measured by the loss of NADPH at OD_{340} for 2mins. Glutathione peroxidase activity assay in GPX kinetic analysis was measured by DTNB method with modification (Drotar et al., 1985). The reaction solution contains 50mM potassium phosphate (pH 7.0), 2mM H_2O_2, 0-0.12mM GSH, 0.06% 5,5-dithio-bis-2-nitrobenzoic acid (DTNB) and 2mL of crude protein extract in a total volume 5mL. The absorbance at OD_{412} was performed for measuring GSH concentration. The activity was recorded as decrease of GSH concentration and calculated the values obtained from the standard curve.

Heat and Salinity stress assay.

Arabidopsis seedlings of WT (Col-0), *vtc1* mutant and overexpression lines of OgCtyAPX1 and AtCytAPx1 were grown on MS medium horizontally, and were subjected to heat stress by submerging them in a water bath maintained at temperature 42° C, as described (Mishra et al., 2018). This was performed for 2 hrs in light/day photoperiod. The seedlings were then returned to growth room and were allowed to recover for 7 days. The number of viable seedlings were quantified and the survival rate was determined. For salt stress, seedlings were transferred to plates containing 150mM NaCl for 2 weeks (Mishra et al., 2018). Fresh weight, phenotypic differences of ten seedling, root length and chlorophyll content were recorded after one week. All experiments were repeated three times.

Enzyme kinetic analysis

50μg purified recombinant OgCytAPX1 protein was used for kinetic analysis assay. The kinetic parameters were calculated based on Lineweaver−Burk plots of the Michaelis−Menten equation,$1/V = Km/Vmax(1/[C] + 1/Km)$.$V$ is the initial reaction rate, Vmax is the maximum reaction, [C] is the substrate concentration, and Km is the Michaelis-Menten constant.

Estimation of H_2O_2, AsA/DHA, and GSH/GSSG

H_2O_2 content in *Arabidopsis* was measured by DMAB–MBTH–POX method (Queval et al., 2008; Veljovic-Jovanovic et al., 2002). 50 mg *Arabidopsis* leaves were ground in liquid nitrogen and the tissue powder extracted in 2 mL 1 M $HClO_4$ including 5% PVP. Homogenates were centrifuged at 12,000x g for 10 min at 4 °C and the supernatant was neutralized by 5 M K_2CO_3 to pH 5.6. The homogenate then was centrifuged at 12,000x g for 1 min to remove $KClO_4$. The sample was incubated with 1 U ascorbate oxidase (Sigma) for 10 min to oxidize ascorbate. The reaction mixture includes 0.05 M phosphate buffer (pH 6.5), 3.3 mM DMAB, 0.07 mM MBTH, and 0.1U horseradish peroxidase (Sigma). The reaction was initiated by adding 50 μL of sample. The change of absorbance at 590 nm was monitored at 25 °C for 5 min, and this value is the total peroxide in samples. To avoid the interference of other peroxides, the samples incubated with 1 U catalase (Sigma) for 10 min, and then followed the same procedures described as above. This absorbance change at OD_{590} is other peroxide, excluding H_2O_2. H_2O_2 contents were subtracted other peroxide from total peroxide, and calculated by the reference to an internal standard (1.5 nmol H_2O_2).

Total AsA, reduced AsA, and total DHA were measured following the method described previously (Gillespie and Ainsworth, 2007). 40 mg plants tissue was extracted

by 6% trichloroacetic acid (TCA) followed by the addition of 10 mM dithiothreitol (DTT) to reduce the pool of oxidized AsA. Total AsA content was measured at OD_{525}. Total AsA (DTT added) and reduced AsA levels (DTT not added) were obtained using this method. Total DHA content was obtained by subtracting the reduced AsA from total AsA.

The levels of GSH and GSSG were assayed by the modified method described previously (Rahman et al., 2006). Tissue sample (0.1 g) was homogenized in 0.6% sulfosalicylic acid solution and then centrifuged at 16,000x g for 10 min at 2–4 °C. The clear supernatant was transferred to a new tube and used for the total GSH assay. In a 96-well microtitre plate, 20 μl of 0.1 M potassium phosphate buffer with 5 mM EDTA disodium salt pH 7.5 (KPE) was placed in a well, and then 20 μl of the sample was added. Next, equal volumes of freshly prepared 5,5-dithio-bis-2-nitrobenzoic acid (DTNB; 0.06% in KPE) and glutathione reductase (GR; 3 units in KPE) solutions were mixed together and 120 μl of the mixture added to each well. A yellow color was obtained after the final reaction. Tube containing the DTNB:GR mixture was covered by aluminum foil to avoid direct exposure to light. After 30 s to allow for the conversion of GSSG to GSH, 60 μl of β-NADPH was added. The absorbance at OD_{412} was performed immediately in a microplate reader, measured every 30 s for 2 min (five readings in total from 0–120 s). The rate of 2-nitro-5-thiobenzoic acid formation (change in absorbance min^{-1}) was calculated. The actual total GSH concentration in the samples was determined by using linear regression to calculate the values obtained from the standard curve.In GSSG assay, 2 μL 2-vinylpyridine (Sigma) was added to 100μl extract and mixed well to derivative GSH. The mixture was reacted for 1 h at room temperature in a hood. Measurement of the derivative samples by the method described as same as total GSH.

The concentration of total GSH and GSSG in samples were determined by using linear regression to calculate values obtained from a standard curve of GSH and GSSG (containing 2 µl 2-vinylpyridine) (Sigma).

Isothermal Titration Calorimetry (ITC)

Binding of GSH to wild-type *OgCytAPX1* and its mutant were measured by ITC with a Nano Isothermal Titration Calorimeter (TA Instruments). Aliquots of 4 µl of 2.0 mM GSH were titrated by injection into protein (0.1 mM in 0.98 ml) in 25 mM phosphate (pH7.0) and 100 mM NaCl. Experiments were carried out at 25 °C with 250 rpm stirring. Background heat from ligand to buffer titrations was subtracted during data processing, and the corrected heat from the binding reaction was used to derive values for the stoichiometry of the binding (n), K_d, apparent enthalpy of binding (ΔH), and entropy change (ΔS). Data were fitted by use of an independent binding model with Launch NanoAnalyze version 2.3.6.

Circular Dichroism (CD) Spectroscopy

Far-UV CD spectra were measured over 190-260 nm wavelength with 20 µM protein sample placed into a 1-mm path length cuvette and recorded on a JASCO J-810 spectropolarimeter (JASCO international Co.) equipped with a Peltier temperature control system (JASCO PTC-423S). All samples were centrifuged at 10,000g for 10 min before analysis. Experiments were carried out at 25 °C. Data processes including baseline subtraction and smoothing were done by Origin.

UV-Visible spectroscopy

Spectra of 5 µM recombinant proteins were placed into a 1-mm path length cuvette and monitored on a UV–visible spectrophotometer (Hitachi U-3010) between 350 and

500 nm (300 nm min^{-1}) in buffer containing 25 mM phosphate (pH7.0) and 100 mM NaCl in absence and presence of GSH at different concentration (from 0.1 to 3.0 mM). Experiments were carried out at 25 °C.

Table S1 The reaction rate of *CytAPX1* against AsA substrate in planta (related to **Figure 2**)

Organisms	K_m value	References
Oncidium	0.626mM	In this study
Theobroma cacao	0.419 μM	(Camillo et al., 2013)
Pea	20μM	(Mittler and Zilinskas, 1991)
Soybean	11 μM	(Dalton et al., 1987)
Palllavicinia	28.7 μM	(Sajitha Rajan and Murugan, 2010)

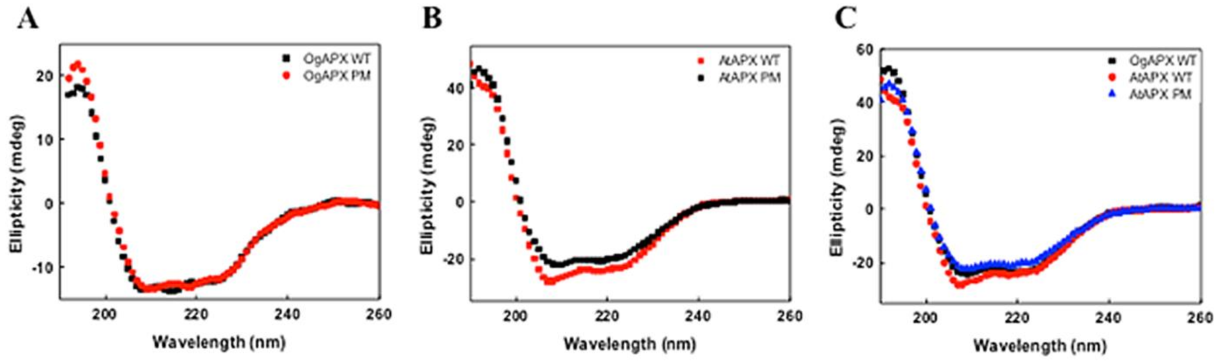

Figure S1 Protein folding comparison of wild-type protein and mutants (related to **Figure 4**).

Far UV circular dichroism spectra of **(A)** Og*CytAPX1* wild type and mutant protein (at 100 μM concentration in 20mM phosphate,pH 7.0 room temperature). **(B)** At*CytAPX1* wild-type and mutant protein (at 200 μM concentration in 200mM phosphate,pH 7.0, room temperature), and **(C)** Og*CytAPX1* wild type, At*CytAPX1* wild type and At*CytAPX1* mutant protein (at 200μM concentration in 20mM phosphate,pH 7.0, room temperature) were monitored over 190-260nm wavelength. The experiments were repeated three times and representative as dotted traces

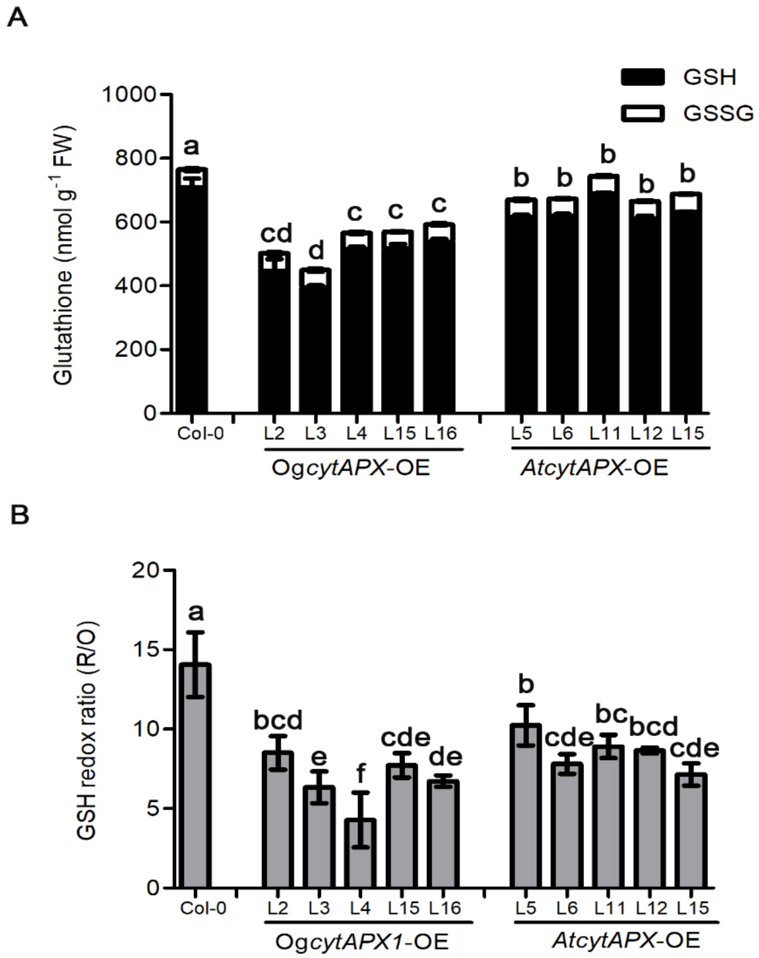

Figure S2 (A) GSH, GSSG content, and (B) GSH redox ratio in *OgCytAPX1-OE* and *AtCytAPX1-OE Arabidopsis* independent lines grown at 22°C (related to **Figure 8**). Error bar indicates SD (standard deviation of the mean (n=30). Statistical significance was analysed by Analysis of variance (ANOVA) with post-hoc test. Different letters indicates significant differences between wild type and transgenic lines according to Fisher's protected LSD test at a significant level of $p<0.05$.
